# Activating low-temperature diesel oxidation by single-atom Pt on TiO_2_ nanowire array

**DOI:** 10.1038/s41467-020-14816-w

**Published:** 2020-02-26

**Authors:** Son Hoang, Yanbing Guo, Andrew J. Binder, Wenxiang Tang, Sibo Wang, Jingyue (Jimmy) Liu, Tran D. Huan, Xingxu Lu, Yu Wang, Yong Ding, Eleni A. Kyriakidou, Ji Yang, Todd J. Toops, Thomas J. Pauly, Rampi Ramprasad, Pu-Xian Gao

**Affiliations:** 1 Department of Materials Science and Engineering & Institute of Materials Science, University of Connecticut, Storrs, CT 06269-3136 USA; 2 College of Chemistry, Central China Normal University, 430079 Wuhan, China; 3 Oak Ridge National Laboratory, Oak Ridge, TN 37932 USA; 4 Department of Physics, Arizona State University, Tempe, AZ 85287 USA; 5 Shanghai Synchrotron Radiation Facility, Shanghai Institute of Applied Physics, Chinese Academy of Sciences, 201800 Shanghai, China; 6 School of Materials Science and Engineering, Georgia Institute of Technology, Atlanta, GA 30332 USA; 7 Umicore Autocat USA Inc., Auburn Hills, MI 48326 USA

**Keywords:** Pollution remediation, Heterogeneous catalysis, Porous materials, Nanowires

## Abstract

Supported metal single atom catalysts (SACs) present an emerging class of low-temperature catalysts with high reactivity and selectivity, which, however, face challenges on both durability and practicality. Herein, we report a single-atom Pt catalyst that is strongly anchored on a robust nanowire forest of mesoporous rutile titania grown on the channeled walls of full-size cordierite honeycombs. This Pt SAC exhibits remarkable activity for oxidation of CO and hydrocarbons with 90% conversion at temperatures as low as ~160 ^o^C under simulated diesel exhaust conditions while using 5 times less Pt-group metals than a commercial oxidation catalyst. Such an excellent low-temperature performance is sustained over hydrothermal aging and sulfation as a result of highly dispersed and isolated active single Pt ions bonded at the Ti vacancy sites with 5 or 6 oxygen ions on titania nanowire surfaces.

## Abstract (web summary)

Supported metal single-atom catalysts face challenges on both durability and practicality. Here, the authors demonstrate that a sustained 90% diesel oxidation conversion at ~160 ^o^C is achieved by single-atom Pt on TiO_2_ nanowire-array integrated catalytic converter.

## Introduction

Effective catalytic oxidation of CO, hydrocarbons (HCs), and nitrogen oxides (NO_x_) at low temperature is sought for ultra-clean and energy-efficient emission control over various energy systems, including both mobile and stationary sources. The exemplary catalytic devices range from diesel oxidation catalysts (DOCs)^1,2^, three-way catalysts (TWCs)^3^, selective catalytic reduction (SCR) of NO_x_ catalysts^4^, to lean NO_x_ traps (LNT)^5^. Developing low-temperature emission control solutions could enable advanced energy and environmental technologies that operate in a more energy-efficient and eco-friendly environment, such as the low-temperature combustion regime engine technology^6^.

In search of low-temperature oxidation catalysts, promising results have been shown using gold^7–9^, metal oxides, such as Co_3_O_4_^10,11^, Co-Cu-Ce mixed oxides^1^, and La-based perovskites^2^. Unfortunately, these catalysts usually are poor in hydrothermal stability and susceptible to sulfur poisoning. Recently, supported platinum-group metal (PGM) single atom catalysts (SACs) have emerged as a new type of low-temperature catalysts with high reactivity and selectivity, due to their high metal dispersion, fewer types of active sites, low-coordination environments, quantum size effects, and enhanced metal-support interactions^12–19^. However, isolated metal atoms are usually mobile and tend to aggregate into clusters or particles during synthesis or reaction. Improving SACs’ stability and maintaining their high activity is one of the grand challenges^20–22^ currently. Recently, Nie et al. reported highly active and stable Pt SACs supported by CeO_2_ that were prepared using the atom trapping method^23^. The catalyst showed good CO oxidation performance with total CO conversion at 148 °C and stability up to 500 °C. However, deactivation was observed after exposure to 800 °C. Meanwhile, the studies on SACs up to now have been limited to model reactions, such as CO oxidation and water gas shift. Little success has been achieved in the supported SACs so far over hydrocarbon oxidation at low temperature. Furthermore, the reported SACs have been limited to lab-scale reactors with a small amount of powders. There has yet to be an evaluation of SACs in field-size catalytic reactors under realistic exhaust conditions, although such a real-world demonstration is necessary to translate the scientific advancement to technological applications.

**Fig. 1:**
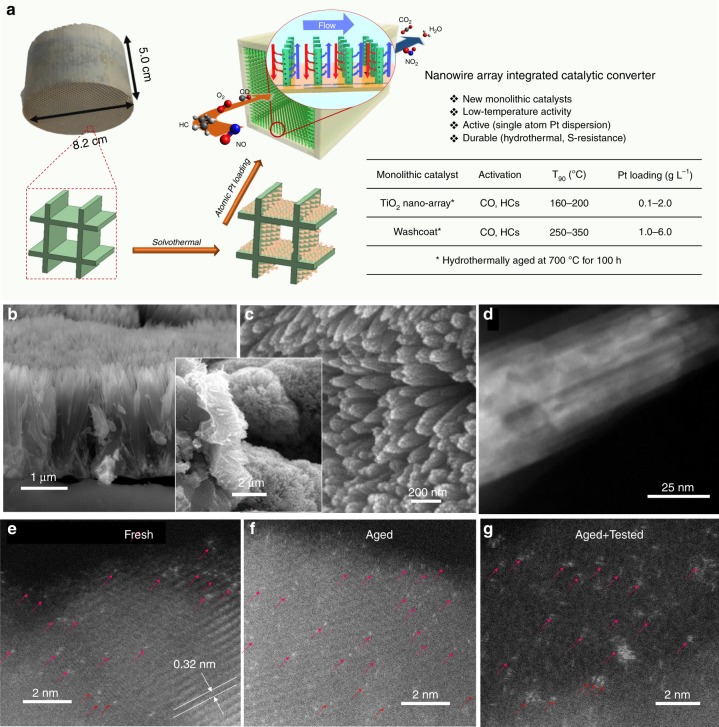
Synthesis and structure of single Pt atoms supported on rutile TiO_2_ nanowire arrays. **a** Schematic illustration of integration process of Pt_1_/TiO_2_ nanowire array forest onto ceramic monoliths and physicochemical and catalytic characteristics of such DOC catalytic converters. **b** Cross-sectional and **c** top view SEM images of rutile TiO_2_ NA on a cordierite honeycomb; inset: low-magnification cross-sectional view of cordierite substrate interface with conformably distributed TiO_2_ nanowire forest. **d** HAADF STEM of a rutile nanowire bundle. The arrays of dark spots on the HAADF STEM image identified the mesoporosity of the TiO_2_ nano-arrays. **e**–**g** ac-HAADF STEM images of Pt_1_/TiO_2_ NA prepared by microwave-assisted dip-coating (0.71 g_Pt_ L^−1^) (**e**) before, (**f**) after hydrothermal aging at 700 °C for 100 h, and (**g**) after hydrothermal aging at 700 ^o^C for 100 h followed by simulated CDC exhaust test treatment. The bright dots on the surface of TiO_2_ are Pt atoms, as pointed by red arrow-heads.

In this work, durable single-atom Pt catalysts are successfully demonstrated to be anchored on titania nanowire array (nano-array, NA) forest integrated on full-size honeycomb substrates, as denoted as Pt_1_/TiO_2_ NA catalyst. As a SAC based monolithic catalyst, it displays a sustained and remarkably high oxidation activity over CO and hydrocarbons at low temperature under simulated diesel exhaust, the industry-defined conditions protocoled by USDRIVE^24^ and under heavy duty diesel (HDD) engine transient dynamic cycles, as illustrated and summarized in Fig. 1a. We demonstrate that the temperature of 90% conversion, *T*_90_, for CO and HCs in the clean diesel combustion (CDC) simulated exhaust evaluation^25^ approaches 160 °C, while using five times less PGM than a benchmark commercial DOC. Notably, the single-atom Pt active sites are highly robust after hydrothermal (HT) aging at 700 °C for 100 h as a result of their fixation at the five-fold coordinated Ti vacancy sites bonded with five or six oxygen ions on titania nanowire surfaces. Such a NA supported Pt SAC retains its nanowire array structure as well as abundant Pt single-atom species on the TiO_2_ nanowire surfaces after the hydrothermal aging and simulated exhaust test.

## Results

### Structure and stability of single-atom Pt on TiO_2_ nanowire arrays

It is noted that pure rutile phase, the most thermodynamically stable among TiO_2_ polymorphs as typical oxidation catalyst support materials^26,27^, is realized in the form of TiO_2_ NA forest in this study. Additionally, the hierarchically structured NA forest allows efficient mass transport and good catalytic performance in supported catalysts in a honeycomb monolithic device form, with advantageous attributes, such as low pressure drop and high surface area^28^. In this work, densely packed forest of mesoporous rutile TiO_2_ NA was successfully grown onto channel surfaces of cordierite honeycombs with various sizes ranging from a size of 0.2 L (63 mm × 63 mm × 50 mm), to a full-size core of 1.2 L (Ф 145 mm × 75 mm). The TiO_2_ loading, determined from inductively coupled plasma mass spectrometry (ICP-MS) measurement, is ~23 wt.% or ~69 g L^−1^. The rutile TiO_2_ NA was grown uniformly with a height of ~3 μm, and arranged in the form of individual nanowire-bundles ~50–100 nm wide (Fig. 1b). The comprised TiO_2_ nanowires are ~10–20 nm wide, as revealed in the high angle annular dark field (HAADF) scanning transmission electron microscopy (STEM) image in Fig. 1d and high resolution transmission electron microscopy (HRTEM) image (Supplementary Fig. 1a). The bundled TiO_2_ nanowires are mesoporous, revealed by arrays of darker spots (3–20 nm in diameter) in Fig. 1d, consistent with the BJH pore size distribution (Supplementary Fig. 2b). The HRTEM lattice image identified an inter-planar lattice spacing of 0.326 nm, matching the {110} planes of rutile TiO_2_, corroborating with the XRD pattern in Supplementary Fig. 1b (JCPDS #88-1175). We note that a thin mesoporous layer of SiO_2_ was also formed during the TiO_2_ growth process. These mesoporous SiO_2_ are formed due to the leaching of Al, Fe, and Mg from cordierite by hydrochloric (HCl) acid at the initial stage of the solvothermal TiO_2_ growth process (Supplementary Fig. 3 and Supplement Note 1).

The rutile TiO_2_ NA integrated cordierite honeycomb has a high BET surface area of ~89.6 m^2^ g^−1^, equivalent to 25,984 m^2^ for a 1 L cordierite honeycomb core. Such a high surface area of the TiO_2_ NA integrated honeycomb is attributed to the unique mesoporous structures of nanowire arrays and partially etched cordierite substrate surface as discussed earlier (Fig. 1d and Supplementary Fig. 2). After Pt loading using a dip-coating method (Method section), the surface area reduces to 45.7 m^2^ g^−1^ (Supplementary Fig. 2b), comparable to the surface area of bench-mark commercial DOC sample (49.8 m^2^/g). After hydrothermal aging at 700 °C for 100 h, the surface area of the sample is further reduced to ~16 m^2^ g^−1^, however with the array structure well retained (Supplementary Fig. 2b). The loss of surface area after both dip-coating and HT aging processes are due to the closure of small pores within TiO_2_ nanowires (<7 nm), as revealed in the pore size distribution measured by N_2_ isotherms and by presence of fewer (dark-spot) pores revealed in the HAADF STEM image of the respective samples (Supplementary Fig. 2b). It is noted that, despite the over 60% decrease of surface area after hydrothermal aging on the Pt_1_/TiO_2_ NA, the superb catalytic oxidation performance has been largely retained. This is due to the remarkable activity and durability found on the well-dispersed and isolated single-atom Pt catalysts anchored on the mesoporous rutile TiO_2_ nanowire surfaces.

During operation, oxidation catalysts may endure mechanical vibrations and high velocity multi-phase exhaust flow such as the vehicular conditions. The associated mechanical stresses might cause the separation or delamination of washcoat layers from the monolithic substrates, resulting in loss of active materials, deactivation of catalysts, and even damage of downstream functional devices^29^. Since the TiO_2_ nano-array forest was “in situ grown” instead of “wash-coated” on the honeycomb monoliths, these nano-array integrated monoliths have an improved adherence over conventional washcoated samples. Evidently, after ultrasonicating at 40 kHz in water bath at 25 °C for 4 h, both fresh and HT aged Pt_1_/TiO_2_ NA integrated monoliths showed little morphology change (Supplementary Fig. 4). A negligible weight loss of ~1% was observed in both fresh and aged nano-array integrated monoliths, as compared to that of >10 % weight loss for the commercial DOC after similar ultra-sonication testing.

Pt was loaded on TiO_2_ nano-arrays, with a volumetric Pt loading of 0.53-1.73 g L^−1^ (0.18–0.58 wt.%, Supplementary Table 1) to prepare functional DOC devices, employing either microwave-assisted dip-coating or wet-incipient impregnation (WII)^12^. Aberration-corrected (ac) HAADF STEM, a powerful tool for discerning individual heavy atoms (e.g., Pt) from lighter supporting atoms (e.g., Ti, O), was employed to investigate the distribution of Pt on the TiO_2_ nano-array. Supplementary Fig. 5 shows ac-HAADF STEM images of Pt supported on TiO_2_ NA prepared using Na-promoted WII method, clearly revealing the well-dispersed Pt single atoms on TiO_2_ nanowire surface. High Pt dispersions of 80% and 74% were determined for the Pt loading of 0.71 and 1.73 g_Pt_ L^−1^, respectively, by H_2_ chemisorption measurement, further confirming the dominant atomically dispersed Pt sites. Interestingly, without any promoter, microwave-assisted dip-coating also results in atomic dispersion of Pt on TiO_2_ nano-arrays shown in Fig. 1e and Supplementary Fig. 6. However, the Pt dispersion of dip-coating samples is lower, 33% and 24% for the Pt loading of 0.71 and 1.73 g_Pt_ L^−1^, respectively, due to a higher amount of Pt nanoparticles dispersed on the adjacent mesoporous SiO_2_ (Supplementary Figs. 3 and 6). Meanwhile, TiO_2_ NA and NW samples with different mesoporosity were prepared under different calcination temperatures and used to look into the support porosity influence on the Pt SAC loading. It is noted that the Pt single-atom distribution is retained in all these different mesoporous TiO_2_ nanowire surfaces, where few to no Pt nanoparticles were observed (Supplementary Fig. 7). This may suggest that the Pt SAC loading is relatively insensitive to the mesoporosity of TiO_2_ NWs. In addition, the mesoporous NA samples maintained a similar specific surface area of 20–25 m^2^ g^−1^ despite the obvious evolution of mesopore size and distribution as the calcination temperature increased from 500 to 900 °C (Supplementary Fig. 8), indicating the excellent thermal stability of these rutile nano-arrays.

**Fig. 2:**
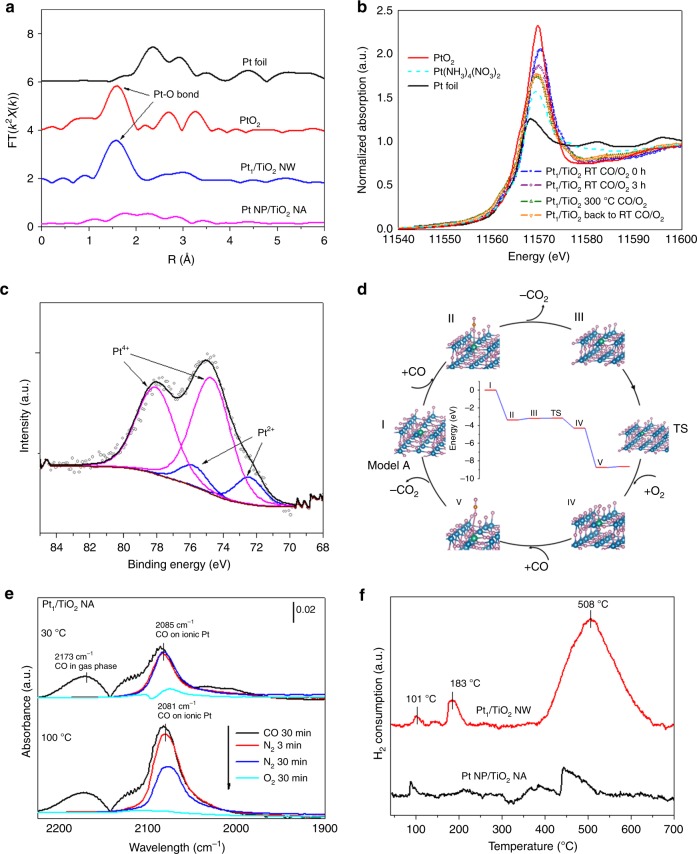
Characterization of the Pt single-atom species on TiO_2_ NW and NA surfaces. **a** k^2^-weighted Fourier-transformed EXAFS spectra of Pt_1_/TiO_2_ NW. Pt NP/TiO_2_ NA, Pt foil, and PtO_2_ are employed as references. **b** Normalized XANES spectra at the Pt L_3_ edge of Pt_1_/TiO_2_ NW under in situ CO oxidation. **c** Core Pt 4 *f* XPS spectrum of the Pt_1_/TiO_2_ NW. **d** The proposed Eley-Rideal mechanism for CO oxidation on Pt_1_/TiO_2_ NA. The reaction cycle shows the structure of intermediates and transient state (TS) of the key elementary steps. The inset shows the calculated energy profile. **e** In situ DRIFT spectra of CO adsorption and oxidation at 30 and 100 °C of Pt_1_/TiO_2_ NA, and **f**, H_2_-TPR profile of Pt_1_/TiO_2_ NW.

It was noted earlier that the single Pt atom catalysts are often not stable under the HT condition due to their tendency to agglomerate, thus hindering their practical applications^30^. Here on the mesoporous rutile TiO_2_ nanowire supports, the atomically dispersed Pt was found to retain its dispersion even after HT aging at 700 ^o^C for 100 h and simulated exhaust treatment (Fig. 1f, g and Supplementary Fig. 9). On both dip-coating and Na-promoted WII samples, these single-atom Pt catalysts retained their dispersion after HT degreening at 700 °C for 4 h (Supplementary Figs. 10 and 11). On the contrary, Pt nanoparticles supported on adjacent SiO_2_ on both samples severely sintered after HT degreening (Supplementary Figs. 10 and 11). To understand the excellent durability displayed here, a series of in situ CO oxidation studies were conducted over the Pt_1_/TiO_2_ SAC catalysts using synchrotron X-ray adsorption spectroscopy (XAS), X-ray photoelectron spectroscopy (XPS), and density functional theory (DFT) computation (Figs. 2a–d). It is found that the strong electrostatic interactions between Pt and TiO_2_ support surface are responsible for the excellent durability in these single Pt atom catalysts^21,22,30^. Specifically, on the TiO_2_ nanowire surfaces, isolated and catalytically active Pt ions are pre-dominantly located at the surface Ti vacancies, and strongly anchored by five or six neighboring O ions, thus exhibiting outstanding hydrothermal durability. Additional studies using in situ diffuse reflectance infrared fourier-transform spectroscopy (DRIFTS) and temperature programmed reduction (TPR) under hydrogen have revealed an outstanding low-temperature catalytic activity of these TiO_2_ nanowire supported SAC catalysts toward CO oxidation (Figs. 2e, f).

### Structure and reactivity of single-atom Pt on TiO_2_ nanowire surface

The in situ XAS studies for CO oxidation were performed on 0.2 wt.% Pt_1_/rutile TiO_2_ nanowires (NW) for understanding the structural characteristics and low-temperature reactivity of the Pt single-atom active sites. TiO_2_ NW powder, collected in the same batch with the synthesis of TiO_2_ NA monolith, was employed as the support to avoid the interference from the cordierite substrates. The fourier transformed-extended X-ray absorption fine structure (EXAFS) spectrum of 0.2 wt.% Pt_1_/TiO_2_ at the Pt *L*_*3*_ edge in r-space shows a dominant peak at 1.6 Å from the Pt–O contribution and very weak peaks at 2.6 and 3.0 Å from either the Pt–Pt or the Pt–Ti contribution (Fig. 2a), confirming that Pt is atomically distributed on TiO_2_ NW surface. Structural parameters extracted from the Pt *L*_*3*_-edge EXAFS fitting, summarized in Supplementary Table 2, indicates that on Pt_1_/TiO_2_ NW, the Pt site has 5.5 Pt–O coordinations, which have an interatomic distance of 1.99 ± 0.02 Å, almost the same as the Pt–O distance in PtO_2_ (2.01 ± 0.02 Å). As shown in Fig. 2b, the white line intensity at ~11570 eV of Pt_1_/TiO_2_ NW in the normalized X-ray Absorption Near-edge Structure (XANES) is between the intensities of Pt(NH_3_)_4_(NO_3_)_2_ and PtO_2_, suggesting that the Pt single atoms have oxidation states in between 2^+^ and 4^+^, corroborating with the XPS analysis that the sample only contains Pt^4+^ and Pt^2+^ (Fig. 2c). During the CO oxidation reaction in lean conditions (2000 ppm CO and 12% O_2_), the overall oxidation state of Pt decreases slightly, but remain in between 2^+^ and 4^+^, indicated by the decrease in the white line intensity of Pt_1_/TiO_2_ NW under the in situ XANES measurements.

DFT calculations were performed to find the most stable configurations of single-atom Pt on TiO_2_ NW surfaces. The binding energy, defined as the energy needed to move a Pt atom from the TiO_2_ surface to vacuum (>10 Å), is used to assess the stability of the Pt atom on the surface. We only considered configurations on rutile TiO_2_ (110) with and without Ti vacancies since the {110} is the dominant surfaces and no O vacancies were detected on TiO_2_ NW (Supplement Note 2). The structures and the corresponding binding energy of Pt are shown in Supplementary Fig. 12. The calculation predicts that the configuration where Pt stays in the five-fold coordinated Ti vacancy (Pt–Ti_V_) is the most stable, with 2.3 eV higher in binding energy compared to that with Pt substituting six-fold coordinated Ti (Pt–Ti_VI_) and at least 1.8 eV higher than other configurations where Pt adsorbed on the TiO_2_ (110). This prediction is in good agreement with XAS fine structure studies. We note that the Pt–Ti_V_ configuration has two models, with (Model A) and without (Model B) O on top (O_top_) of Pt. In model A, Pt is fully oxidized (Pt^4+^) and has six Pt–O coordinations including four in-plane Pt–O_i_, one Pt–O_sub,_ and one Pt–O_top_ where O_i_ is in-plane O, and O_sub_ is O from the sub-surface layer. The Pt–O_top_ is a weak bond and can be easily activated, thus this bond is absent in the partially oxidized form (Pt^2+^) in model B, leaving 5 coordinations on the Pt sites. The binding energies of Pt in the Models A and B are close to each other (10.44 eV vs. 11.09 eV), suggesting the likely co-existence of both Pt^4+^ and Pt^2+^on the TiO_2_ NW surface, therefore, the average coordination number of Pt is between 5 and 6, in agreement with the experimental result from both fitting EXAFS curve (Supplementary Table 2) and XPS analyses (Fig. 2c and Supplementary Table 3).

It is noted that the white line intensity of Pt_1_/TiO_2_ NW decreases in the CO/O_2_ mixture even at room temperature, suggesting isolated Pt atoms supported on TiO_2_ NW may catalyze room-temperature CO oxidation. We employed DRIFTS to obtain mechanistic insights into low-temperature CO oxidation reactivity of Pt_1_/TiO_2_ NA. Fig. 2e shows the in situ DRIFT spectra of CO adsorption and oxidation at 30 and 100 °C of Pt_1_/TiO_2_ NA. After CO is adsorbed on Pt_1_/TiO_2_ NA, quasi-symmetrical IR bands at 2085 and 2081 cm^−1^ are observed for the spectra collected at room temperature and 100 °C, respectively, and ascribed to CO linearly adsorbed on isolated ionic Pt^δ+^ sites^19,21,22^. The IR band at 2085 cm^−1^ shows a reduction of 80% in peak area after O_2_ was introduced for 10 min at room temperature, suggesting CO is readily oxidized at room temperature on Pt_1_/TiO_2_ NA. All adsorbed CO is oxidized when the temperature increases to 100 °C as the IR band at 2081 cm^−1^ vanishes with the introduction of O_2_. These evidences clearly demonstrate low-temperature reactivity of Pt_1_/TiO_2_ NA.

DFT calculations were also performed to elucidate the high catalytic activity for CO oxidation of Pt_1_/TiO_2_ NA and NW. CO can be oxidized over supported Pt catalysts in three ways, namely Langmuir-Hindshelwood (LH), Mars-van Krevelen (MvK), and Eley-Rideal (ER) mechanisms^31–33^. On Pt_1_/TiO_2_ NA and NW, the ER mechanism was found to be favored due to a very low DFT derived activation energy of ~ 0.2 eV (~19 kJ mol^−1^), in good agreement with the experimentally measured activation energy of 22.7 kJ mol^−1^ (Supplementary Fig. 13). The ER catalytic cycle and the corresponding calculated energy profile of CO oxidation on Pt_1_/TiO_2_ NA is shown in Fig. 2d. Model A with a Pt^4+^ reactive center was employed as the starting configuration (Intermediate I). CO reacts with the O on top of Pt via the ER mechanism with an activation energy of 0.2 eV and exothermicity (∆H) of –3.6 eV, leaving one free coordination on Pt (Intermediate II). This free coordination is then filled by adsorption of an O_2_ molecule (Intermediate III). The O–O bond of O_2_ adsorbed on the Pt site can be easily activated when another CO coordinates with one O atom at the other end (Intermediate IV). The cycle is completed with the release of CO_2_. It is worth noting that the LH mechanism is usually dominated for supported Pt nanoparticle catalysts (except for Pt/CeO_2_ case where MvK is dominant^21^), which involves adsorption of CO and dissociative adsorption of O_2_ on metal active sites, followed by the reaction of adsorbed CO and O species to form CO_2_^33^. A modified LH scheme was proposed for CO oxidation on Pt single atoms supported on FeO_x_ (Pt_1_/FeO_x_), in which CO adsorbing on the Pt atom reacts with O from FeO_x_ support^15^. Meanwhile, the LH-based mechanisms are energetically unfavorable on Pt_1_/TiO_2_ NA due to a high activation energy of ~1.8 eV (174 kJ mol^−1^) for the dissociation of the adsorbed O_2_ molecule with the help of the Pt site and one surrounding Ti site (Supplementary Fig. 14).

The redox property of Pt_1_/TiO_2_ NW was studied using H_2_ temperature-programmed reduction (H_2_-TPR) (Fig. 2f). In the H_2_-TPR profile of Pt_1_/TiO_2_ NW, the peaks at 103 and 508 °C are assigned to the reductions of isolated Pt atoms and bulk TiO_2_, respectively. We observed an additional H_2_-consumption peak at 186 °C for Pt_1_/TiO_2_ NW, which is absent in the profile of Pt NP/TiO_2_ NA. This peak can be assigned to the reduction of TiO_2_ induced by isolated Pt atoms. It has been reported that the presence of single-atom Pt can lower the reduction temperature of metal oxide support due to the strong metal-support interaction^15^. The enhanced reducibility of Pt_1_/TiO_2_ NA compared to other TiO_2_ based-DOCs might allow excursions into fuel-rich and fuel-lean conditions although more thorough evaluation is necessary.

### Low-temperature diesel oxidation over single-atom Pt on TiO_2_ nanowire arrays

To evaluate their catalytic oxidation activity, the Pt_1_/TiO_2_ NA monoliths were tested under simulated engine exhausts based on clean diesel combustion (CDC) and low-temperature diesel combustion (LTC-D) protocols developed by US DRIVE^24^. The samples were tested at a high gas hourly space velocity (GHSV) of 60,000 h^−1^ to assess their close-to-reality performance under these protocoled conditions. We note that in both conditions, the composition of total hydrocarbons (THC) on a C_1_ basis in the exhausts is ~56% C_2_H_4_, 33% C_3_H_6_, and 11% C_3_H_8_. Samples were hydrothermally degreened at 550 ^o^C for 4 h and at 700 ^o^C for 4 h for evaluation in the CDC and LTC-D conditions, respectively, representing a ~5000 mile-on-road condition.

**Fig. 3:**
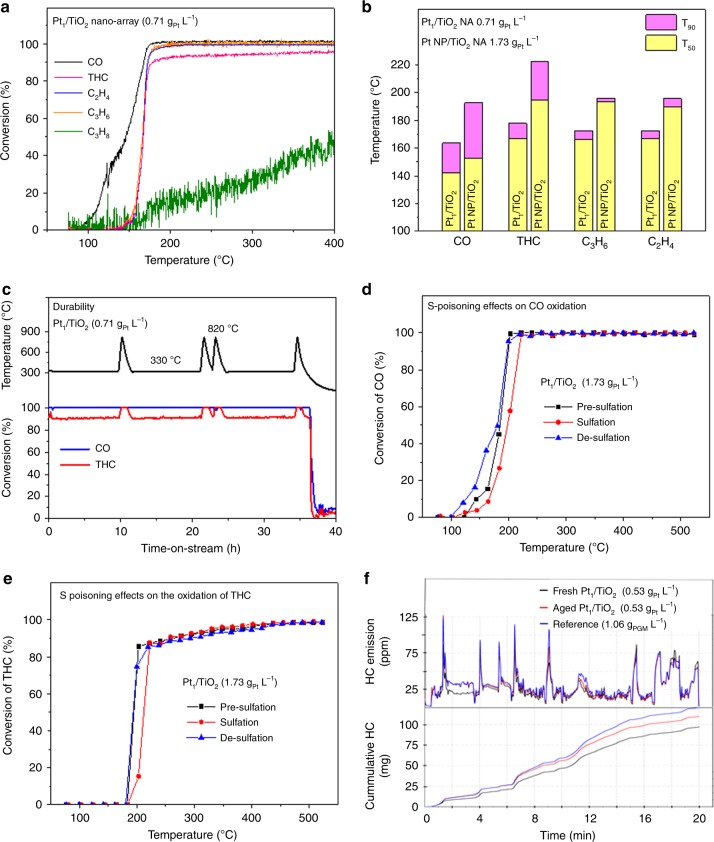
Diesel oxidation performance of Pt_1_/TiO_2_ NA integrated monoliths. **a** Light-off curves for Pt_1_/TiO_2_ NA (0.71 g_Pt_ L^−1^) in the CDC simulated exhaust. **b** Comparison of the DOC activity in the CDC simulated exhaust between P_1_/TiO_2_ NA (0.71 g_Pt_ L^−1^) and Pt NP/TiO_2_ NA (1.73 g_Pt_ L^−1^). **c** Durability of the Pt_1_/TiO_2_ NA in the simulated exhaust. **d**, **e** Sulfur-poisoning effects on the DOC activity of Pt_1_/TiO_2_ NA (1.73 g_Pt_ L^−1^) in the LTC-D simulated exhaust. **f** Transient (upper panel) and cumulative (lower panel) THC emission in the transient gas conditions mimicking a HDD FTP as running on a HDD certified 2010 Cummins ISB (6.7 L) 320 hp engine for fresh (blue) and aged (red) Pt_1_/TiO_2_ NA (0.53 g_Pt_ L^−1^). A commercial DOC monolith with double PGM loading (1.06 g L^−1^) was employed as the reference.

Fig. 3a shows the light-off curves for Pt_1_/TiO_2_ NA with Pt loading of 0.71 g L^−1^ in the CDC simulated exhaust. The T_90_ temperatures of CO, C_2_H_4_, C_3_H_6_, and THC are 164, 172, 173, and 178 °C, respectively, all well below 200 ^o^C, at which most catalyst systems in today’s vehicles are ineffective^34^. For benchmark, we employed a commercial DOC sample with PGM loading of 4.66 g L^−1^, obtained from a brand new heavy-duty pick-up truck (year model 2012). The temperature T_90_ for THC of Pt_1_/TiO_2_ NA is ~172 °C lower than that of the commercial DOC (350 °C), despite ~5 times less PGM loading (Supplementary Fig. 15). On Pt_1_/TiO_2_ NA, both ethylene and propylene oxidation occur almost simultaneously. On the other hand, the oxidation of ethylene, the main hydrocarbon component in the simulated exhausts (56%), on the commercial DOC sample is sluggish, leading to the slow conversion of THC and consequently a much higher T_90_ than that of Pt_1_/TiO_2_ NA. Additionally, the conversions of both C_3_H_6_ and C_2_H_4_ on the Pt_1_/TiO_2_ NA integrated monoliths quickly reach more than 99 % with no plateau in the high conversion region that was observed in the commercial DOC, suggesting that the catalytic reaction kinetics over Pt_1_/TiO_2_ nano-array are not mass-transfer limited even at a high space velocity of 60,000 h^−1^^35^.

A secondary function of a DOC catalyst is to oxidize NO to NO_2_ to help downstream functionalities such as NO_x_ selective catalytic reduction (SCR) catalyst and regeneration of diesel particulate filters^1,2^. In this regard, the Pt_1_/TiO_2_ NA also outperforms the commercial sample, with a maximum NO-to-NO_2_ conversion of 47% for the Pt/TiO_2_ nano-array at a low temperature 291 ^o^C as compared to 27% at 427 ^o^C for the commercial DOC (Supplementary Fig. 15).

To evaluate and validate the practical diesel oxidation reactivity of isolated Pt atom active sites, we employed Pt-nanoparticle (NP) supported on mesporous SiO_2_ monolith and TiO_2_ NA monolith (Supplementary Fig. 16) as two references. These reference catalysts were prepared by etching of cordierite honeycombs followed by Pt dip-coating, and atomic layer deposition of Pt over TiO_2_ NA monolith^28^, respectively. It is noted the low-temperature DOC activity of Pt NP/TiO_2_ nano-array is good, but inferior to Pt_1_/TiO_2_ NA, despite a 2.5-time higher PGM loading (Fig. 3b). The temperatures *T*_90_ of Pt NP/TiO_2_ NA for CO, C_3_H_6_, C_2_H_4_, and THC are 193, 196, 196, and 223 °C, respectively, ~23–45 °C higher than that of Pt_1_/TiO_2_ NA. When using a Pt NP loading of 0.71 g_Pt_ L^−1^ on the etched cordierite substrate that spontaneously created a layer of mesoporous SiO_2_ support, similarly inferior catalytic oxidation performances were observed over CO and C_3_H_6_, with ~35–45 °C higher *T*_90_ temperatures than those of the Pt_1_/TiO_2_ NA sample of the same Pt loading (Supplementary Fig. 16c). This evidence clearly illustrates the improved reactivity for CO and HC oxidation of isolated Pt atoms, compared to Pt NP active sites although direct turnover frequency (TOF) measurements are difficult or impossible with these steep light-off curves and under the complex flow conditions.

To evaluate the durability of Pt_1_/TiO_2_ NA during reactive conditions, the catalyst was tested under simulated exhaust at 330 ^o^C with cyclic exposures to 820 °C for 10 min (Fig. 3c). No deactivation was observed of the catalyst with the conversions of CO and THC remaining at 100% and >90%, respectively, during a ~36 h evaluating period. The excellent durability of Pt_1_/TiO_2_ NA is attributed to the stable atomically distributed Pt sites, which even retained after hydrothermal aging at 700 °C for 100 h and the CDC simulated exhaust treatment, as revealed in the single-atom structural analyses earlier (Fig. 1f, g).

Sulfur-containing compounds, present in most vehicle fuels and lubricants, can be oxidized to SO_2_ during combustion, interacting with and deactivating PGM supported catalysts^36–38^. Therefore, it is necessary to test sulfur-poisoning effects on Pt_1_/TiO_2_ NA for the practical DOC applications. The sample Pt_1_/TiO_2_ NA with Pt loading of 1.73 g L^−1^ was chosen for the USDRIVE’s testing protocol for sulfur tolerance based on the LTC-D simulated exhaust^24^. Under the LTC-D condition, this sample performed better than the commercial DOC sample (5 ^o^C lower in T_90_ for THC oxidation and much higher max NO-to-NO_2_ conversion (71% vs. 25%)) despite 2.7 times lower in PGM loading (1.73 vs 4.66 g L^−1^) (Supplementary Fig. 17). After an exposure to 1 g_sulfur_ L^−1^ of SO_2_, the sample showed only a slight deactivation for CO and unsaturated HC with a *T*_90_ increase of ~19 °C (Fig. 3d, e and Supplementary Fig. 18). Interestingly, low-temperature catalytic propane oxidation was improved, likely due to the formation of interfacial sulfate species that facilitate propane chemisorption on Pt^36^, thus decreasing *T*_90_ of THC by 9 °C (Fig. 3e and Supplementary Fig. 18). Under simulated exhaust, propane contributes to 11% of THC and propane oxidation often starts after unsaturated HCs. Therefore, *T*_90_ of THC of a DOC is largely dependent on low-temperature propane oxidation activity, as reflected by increased *T*_88_ but decreased *T*_90_ of THC after S-poisoning (Supplementary Fig. 18). After de-sulfation in H_2_ stream, the oxidation catalytic activity for CO was even better and that for unsaturated HCs was almost fully recovered.

To demonstrate the scalability for practical conditions, a full-size Pt_1_/TiO_2_ NA monolith with a Pt loading of 0.53 g L^−1^ and dimensions of Ф 5 cm × 7.5 cm was evaluated under highly transient feed gas conditions, mimicking a heavy-duty diesel (HDD) federal test procedure (FTP) as running on a HDD certified 2010 Cummins ISB (6.7 L) 320 hp engine. The transient feed gas composition is listed in Supplementary Fig. 19. The Pt_1_/TiO_2_ NA sample was aged at 650 °C for 100 h in 10% steam/air flow to a representative end of life condition for a North American HDD application. Fig. 3f and Supplementary Fig. 20 show the transient response and cumulative emission of THC and CO, respectively, for the fresh and aged Pt_1_/TiO_2_ samples. The Pt_1_/TiO_2_ samples do not show reactivity during the first 200 s because of low temperature, high exhaust flow rate, and high concentration of HCs and CO. When the temperature increases, both fresh and aged samples showed good activity despite the drastic changes in both concentration and flow rate of emissions. It is noted that the HC oxidation activity of the aged sample is almost as good as the fresh sample, with only a small difference observed in the transient and cumulative HC emissions. The performance of the aged sample is mainly due to the stable single-atom Pt sites. As revealed by HAADF STEM (Fig. 1f, g and Supplementary Fig. 9), we observed a high density of isolated Pt atoms on the surface of TiO_2_ nanowires even after hydrothermal aging at 700 °C for 100 h followed by the simulated exhaust treatment. However, some small pores (<7 nm) are closed during hydrothermal aging, as indicated by HAADF STEM and N_2_ isotherms, thus access is inhibited on a portion of Pt sites (Supplementary Fig. 2). Such loss of Pt active sites leads to slow response to CO at high flow rate and concentration (at 500, 930, and 1100 s), and consequently an increase in the CO emission.

## Discussions

The remarkable low-temperature performance of the isolated Pt single atoms supported on TiO_2_ nano-arrays can be attributed to the combination of atomically dispersed Pt and the unique structure of long-range ordered mesoporous TiO_2_ arrays. Single Pt atoms clearly show better diesel oxidation activity than the Pt NP counterpart, as demonstrated in the reactivity comparison under the simulated CDC exhaust conditions (Fig. 3b and Supplementary Fig. 15). First, the enhanced low-temperature reactivity of Pt single-atom catalyst is explained by the distinct electronic structures that modify interaction with adsorbate molecules or by the involvement of supports that may alter reaction pathways^15,18^. The LH and MvK mechanisms are often reported for supported Pt catalysts. However, low-temperature CO oxidation on Pt_1_/TiO_2_ NA favors the ER mechanism with very low activation energy. Second, the introduction of macro-pores in the mesoporous NW structures improves mass transport properties^39,40^. In the Pt_1_/TiO_2_ NA, the space between the mesoporous nanobundles acts as a large channel for fast transport of the reactants along the bundles before the reactants diffuse into the mesoporous network to the Pt active sites (Fig. 1a). Well-spaced support also helps to inhibit sintering of Pt, contributing to the hydrothermal robustness. The Pt_1_/TiO_2_ NA structures, which combine durable and reactive single Pt atoms and hierarchically porous NA support, are expected to have broader applications in the field of catalysis and materials science.

In summary, we demonstrate a single-atom Pt catalyst anchored on a highly stable mesoporous rutile titania nanowire array forest grown in field-size honeycomb monolith. With over 80% PGM usage reduction compared to that of the commercial benchmark, such a single-atom Pt catalyst has displayed remarkable oxidation activities for CO and hydrocarbons with 90% conversion at temperatures as low as ~160 °C under simulated exhaust conditions. Such excellent low-temperature activities are sustained over hydrothermal aging and sulfation as the active single-atom Pt species retain on the stable titania nanowire arrays, as a result of a strong electrostatic interaction between Pt single-atom species and TiO_2_ nanowire surface. A simple and cost-effective solvothermal process in conjunction of dip-coating and impregnation methods provides a viable strategy for scalable manufacturing of durable Pt single-atom catalysts on the nano-array-integrated monoliths.

## Methods

### Catalyst preparation

TiO_2_ nanowire arrays are grown on ceramic monoliths via a nonpolar solvent/hydrophilic solid substrate interfacial reaction under hydrothermal conditions^41,42^. Typically, the washed and TiO_2_ seeded cordierite honeycomb substrates with size up to 7.5 cm × 7.5 cm × 5 cm are placed within a sealed Teflon reactor (1 L), containing 500 mL of a non-polar solvent, 50 mL of a Ti (IV) alkoxide precursor, 5 mL of titanium tetrachloride (1 M in toluene), and 50 mL of hydrochloric acid (37 wt.%). At room temperature, titanium (IV) precursors and water are separated since the Ti precursors are dissolved in the nonpolar solvent. Under hydrothermal conditions, water diffuses away from the high-energy water/nonpolar solvent interface to the hydrophilic TiO_2_ nuclei on ceramic wall, where water reacts with Ti precursors, resulting in growth-crystallization of TiO_2_. The presence of Cl^−^ anions is crucial for the anisotropic growth, as they tend to absorb on the rutile (110) plane, thus inhibiting further crystal growth of this plane. After being taken out for the reactor, all the samples are sonicated in acetone, ethanol, and water for 3 h to remove organic compounds and Cl^−^ residues from the synthesis before Pt loading.

Microwave-assisted dip-coating and Na-promoted wet incipient impregnation methods are employed to load Pt on the TiO_2_ nano-arrays. For the dip-coating method, the substrate is first submerged into the diluted Pt precursor solution (0.5 mg Pt precursor per mL). It is taken out and blow-dried using compression air stream. The substrate is then dried in a microwave oven for 1–3 min. These steps are repeated until appropriate amount of the metal precursor is absorbed. Finally, the sample is calcined in air at 500 °C for 2–4 h with a ramp rate of 2 ^o^C min^−1^. In addition, a following 1-h stay at a different calcination temperature such as 700, 800, and 900 °C has been adopted to look into the induced porosity and surface area change and its effect on the single-atom loading and the catalyst stability. For impregnation, the substrate is submerged into diluted Pt precursor (50 μg mL^−1^) and NaOH (atomic ratio Na/Pt is 10/1), and aged for 12 h at 80 °C. The solution is then evaporated in open air at 50 ^o^C until dry. During evaporation, the substrate is rotated every 15–20 min to enhance uniformity. Finally, the sample is dried at 150 °C for 12 h and calcined in air at 500 °C for 4 h with a ramp rate of 2 °C min^−1^.

To evaluate their hydrothermal stability in the probe reactions (CO and C_3_H_6_ oxidation) and the simulated exhaust (clean diesel combustion, CDC; and low-temperature combustion-diesel, LTC-D) tests, the monolithic catalysts are aged in a flow of 12% O_2_, 6% CO_2_, 6% H_2_O in N_2_ at 700 °C for 100 h. In the transient engine tests, the sample is aged in a flow on 10% H_2_O in air at 650 °C for 100 h. These aging conditions are chosen to represent a North America heavy-duty diesel application.

### Catalytic activity evaluation

The catalytic activity measurements were performed in a continuous flow reactor equipped with a gas chromatography. The monolithic catalysts (5 × 5 channels × 2.5 cm long) were placed in a tubular quartz reactor with a surrounding electrical heating coil. The inlet temperature was measured using a 0.16-mm K-type thermocouple, which was fixed at <5 mm in front of the monolith. The utilization of the small thermocouple (0.16 mm) is to prevent disruption of gas flow. Typically, the catalytic activity of each sample was evaluated through total six reaction cycles, in which the inlet temperature was ramped up from 100 to 525 °C at 2 °C min^−1^ and naturally cool down to 100 °C before next testing cycles. The feed gas mixtures were controlled by mass flow controller before entering the blender and then the reactor at a gas hourly space velocity of 30,000 h^−1^_._ The feed gas always contains 12% O_2_ to mimic the O_2_ concentration in the CDC and LTC-D simulated exhaust, but varies CO and C_3_H_6_ concentrations (also were chosen to reflect the CO and total hydrocarbon concentration in the CDC and LTC-D simulated exhausts) in each cycle such that: cycle 1 and 2 contain 2000 ppm CO and 1000 ppm C_3_H_6_; cycle 3 contains 2000 ppm CO; cycles 4 contains 1000  ppm C_3_H_6_; cycle 5 contains 500 ppm C_3_H_6_; and cycle 6 contains 500 ppm CO and 500 ppm C_3_H_6_; all are balanced by N_2_. Sample Array-50-D was evaluated in an additional sequence (cycle 3→4→5→6→1→2), showing negligible difference in the catalytic activity, thus confirming the sequence of the tests does not affect the catalytic activity of TiO_2_ nano-array supported Pt catalysts.

The catalytic diesel oxidation activity of catalysts under simulated exhaust conditions was evaluated according to the protocol developed by the Advanced Combustion and Emission Control (ACEC) Technical Team of USDRIVE. The light-off measurements were conducted on a customized plug-flow reactor system. Micro-cores of catalyst samples were cut and loaded into cylindrical quartz tubing using a wrapping in order to ensure no gas could bypass the catalyst channels. The catalyst temperature was monitored using two thermocouples, one measuring the inlet temperature while located ~2 cm from the catalyst front, the other measuring the mid-catalyst temperature and located from the rear into the middle of the central channel of the micro-core. Space velocity was kept at 60,000 h^−1^ throughout all the tests. Gas concentrations were determined per ACEC Tech Team (USDRIVE) protocol. The composition of the “LTC-D” simulated exhaust is [O_2_] = 12%, [H_2_O] = 6%, [CO_2_] = 6%, [H_2_] = 400 ppm, [CO] = 2000 ppm, [NO] = 100 ppm, [C_2_H_4_] = 833.5 ppm, [C_3_H_6_] = 333 ppm, [C_3_H_8_] = 111 ppm, and Ar balance. The composition of the “CDC” simulated exhaust is [O_2_] = 12%, [H_2_O] = 6%, [CO_2_] = 6%, [H_2_] = 100 ppm, [CO] = 500 ppm, [NO] = 200 ppm, [C_2_H_4_] = 389 ppm, [C_3_H_6_] = 233.5 ppm, [C_3_H_8_] = 51.7 ppm, and Ar balance. Water was introduced via argon flow through a bubbler and heated lines held at 200 °C. Product gas stream was measured via MKS FTIR. Experiments were conducted first with a degreening step in only the O_2_, CO_2_, and H_2_O components of the gas stream at 700 °C for 4 h for LTC-D and 550 ^o^C for 4 h CDC protocols, followed by an evaluation step from 100 to 500 °C at a rate of 2 °C min^−1^. Besides the regular simulated exhaust test, catalyst samples were also treated using the following simulated exhaust test treatment protocol to look into the single Pt atom species fate before and after experiencing the treatment on the Pt/TiO_2_ NA samples with both fresh state and hydrothermal aged condition. CDC simulated gas treatment: 12% O_2_ + 6% H_2_O + 6% CO_2_ + 100 ppm H_2_ + 500 ppm CO + 200 ppm NO + 1400 ppm HCs, with a ramp rate of 5 °C min^−1^ from room temperature to 500 °C, and then keeping at 500 °C for 1 h.

Sulfur-poisoning effects on the catalyst performance were evaluated following USDRIVE’s protocol. The catalyst was exposed to 5 ppm SO_2_ added to the full simulated exhaust at a space velocity of 30,000 h^−1^ and 300 °C catalyst inlet temperature for 5 h. A total sulfur exposure level of 1 g sulfur per liter of catalyst was estimated following this exposure condition. Poisoning was conducted following the pretreatment in which the catalyst was annealed at 600 ^o^C for 20 min before cooled down to 300 °C in 12% O_2_, 6% H_2_O, 6% CO_2_, and N_2_ balance. After sulfur exposure, SO_2_ was removed from the feed and the sample cooled to 100 °C at which point the catalyst performance was evaluated. For de-sulfation, the catalyst was annealed at 700 °C in 3% H_2_ and 1% CO in N_2_ for 30 mins, followed by calcination in air at 500 °C for 2 h.

The Pt/TiO_2_ nano-array sample with a Pt loading of 0.53 g L^−1^ and dimensions of Ф 2″ × 3″ were tested under highly transient feed gas conditions mimicking a heavy-duty diesel (HDD) federal test procedure (FTP) as running on a HDD certified 2010 Cummins ISB (6.7 L) 320 hp engine. The characteristic of the transient feed gas is provided in Supplementary Fig. 19. The Pt/TiO_2_ nano-array and reference samples were aged at 650 °C for 100 h in 10% steam/air flow to a representative end of life condition for a North American HDD application.

### Catalyst characterization

The X-ray absorption fine structure data was collected at the BL14W1 station at the Shanghai Synchrotron Radiation Facility (SSRF). The storage ring of SSRF was operated at 3.5 G eV with a maximum current of 260 mA. Pt foil and PtO_2_ were used as reference samples. Using Si (111) double-crystal monochromator, the data collection was carried out in fluorescence mode for Pt *L*_*3*_-edge with a 7-element detector Ge solid state detector. The energy was calibrated according to the absorption edge of Pt foil (edge energy: 11564 eV).

The EXAFS data was analyzed and fitted using the ATHENA module in the IFEFFIT software packages. Each *k*^2^-weighted EXAFS spectrum was processed by a two-step procedure: (i) subtraction of post-edge background from overall absorption; and (ii) normalization with respect to the edge-jump step. The *k*^2^-weighted χ(k) data of Pt *L*_*3*_-edge were then Fourier transformed to real (*R*) space through a hanning windows (*dk* = 1.0 Å^−1^) in order to separate the EXAFS contributions from different coordination shells. To determine the quantitative structural parameters around central atoms, the ARTEMIS module of IFEFFIT software packages was employed for least-squares curve parameter fitting.

Coordination number of model samples (Pt foil) was fixed as the nominal value. The obtained *S*_*0*_^*2*^ of Pt foil was 0.80. It was fixed in the subsequent fitting of Pt *L*_*3*_-edge date. The fitted ranges for *k* and *R* spaces were selected to be *k* = 3–10.3 Å^–1^ (*k*^2^ weighted) with *R* = 1.0–2.2 Å (Pt_1_/TiO_2_ NW) or 1.4–3.2 Å (Pt NP/TiO_2_ NA).

To conduct the H_2_ temperature programmed reduction (TPR), the samples were degassed in helium at ambient temperature for 20 min. TPR condition: 10% H_2_, balance Ar, 50 ml min^−1^, 10 °C min^−1^. To determine the surface area and pore size distribution of the catalysts, N_2_ adsorption–desorption isotherms and H_2_ chemisorption were carried out using a Micromeritics ASAP 2020 Physisorption analyzer at liquid nitrogen temperature. Brunauer–Emmett– Teller (BET) method and Barret–Joyner–Halenda (BJH) method were used to calculate the specific surface area and pore size, respectively. The samples to be measured were degassed at 350 °C for 4 h in vacuum before the measurement of N_2_ adsorption–desorption isotherm.

The morphology and structure of catalysts were characterized by electron microscopies. Scanning electron microscopy images were taken using FEI Teneo low vacuum SEM and a JEOL 6335F field emission SEM, operating at 10–20 kV. Transmission electron microscopy (TEM) including both bright field and high angular annual dark field (HAADF) images and energy-dispersive X-ray spectroscopy for composition distribution were taken using a FEI Talos STEM and a Tecnai F30 STEM. The aberration-corrected HAADF STEM images were obtained on two STEM/TEM systems: (1) a JEOL JEM 2200FS, attached with a CEOS (Heidelburg, Germany) probe corrector, with a nominal image resolution of 0.07 nm; and (2) a JEOL JEM-ARM200F, equipped with a CEOS probe corrector, with a guaranteed resolution of 0.08 nm. The chemical states of the TiO_2_ and Pt/TiO_2_ nanowire samples were characterized using a Thermo K-Alpha X-ray photoelectron Spectrometer with an Aluminum K-Alpha 1.486 KeV source. X-ray diffraction (XRD) patterns of the samples were collected by a Bruker D2 Phaser using Cu Kα radiation (*λ* = 0.15418 nm) operated at 40 kV and 40 mA. The XRD data were recorded for 2θ values from 10° to 80° with an interval of 0.02°.

H_2_ chemisorption was used to determine the platinum dispersion (D_Pt_) at 35 °C on a Micromeritics ASAP 2020 analyzer. H_2_ reduction treatment of samples was conducted at 300 °C for 2 h, followed by the outgassing at 150 °C for 2 h prior to chemisorption experiment. A double isotherm method was used to determine the H_2_ uptake: first isotherm revealed the total hydrogen consumed (uptake), (HC)_T_, while the second one, acquired after evacuation for 2 h showed the “reversible” or weakly adsorbed hydrogen, (HC)r. The amount of “irreversible” or strongly adsorbed hydrogen, (HC)i, was determined by calculating the isotherm difference. The pressure range of isotherms was controlled at 0–12 kPa, where the extrapolation to zero pressure was used to measure gas uptake. The D_Pt_ was finally calculated by an atomic (HC)i/Pts = 1 ratio, where Pts implies a Pt atom on surface^43^.

To evaluate the mechanical stability of TiO_2_ nano-array integrated cordierite honeycomb samples, a sonication experiment was conducted on both TiO_2_ and Pt/TiO_2_ nano-array samples before and after hydrothermal aging at 700 ^o^C for 100 h. The samples were sonicated in water at 40 kHz for 3 h and weighed before and after the sonication. Little morphology change (in SEM) was observed and the weight loss after the sonication process is less than 1 wt. % for both fresh and aged samples, indicating the sound honeycomb structure integrity and the superior adhesion of TiO_2_ and TiO_2_/Pt nano-arrays on the honeycomb monolith channel walls.

Density Functional Theory (DFT) calculations were performed as implemented in the Vienna Ab initio simulation package^44,45^, employing the Perdew, Burke and Ernzerhof exchange-correlation functional^46^. Models for rutile TiO_2_ (110) surfaces contain two layers along the y direction followed by a vacuum slab of more than 20 Angstrom. The (110) surface was constructed by repeating the cell of dimension 13.16 Å × 11.88 Å along the *x* and *z* directions, respectively. With this model, a k-point mesh of 2 × 1 × 2 and a plane-wave energy cutoff of 400 eV were used. As systems to be modeled involve isolated molecules like CO_2_ and O_2_, the long-range van der Waals interactions was included into the calculations using the scheme of Grimme^47^. Reaction pathways were searched using the nudge-elastic band method by Henkelman and Jónsson^48^.

## Supplementary information

Supplementary Information

## Data Availability

All data generated or analyzed during this study are included in this published article (and its supplementary information files).

## References

[1] Binder AJ (2015). Oxidation over a ternary oxide catalyst with high resistance to hydrocarbon inhibition. Angew. Chem..

[2] Kim CH, Qi G, Dahlberg K, Li W (2010). Strontium-doped Perovskites rival platinum catalysts for treating NOx in simulated diesel exhaust. Science.

[3] Mellor JR (2002). The application of supported gold catalysts to automotive pollution abatement. Catal. Today.

[4] Li J, Chang H, Ma L, Hao J, Yang RT (2011). Low-temperature selective catalytic reduction of NOx with NH3 over metal oxide and zeolite catalysts—a review. Catal. Today.

[5] Liu G, Gao P-X (2011). A review of NOx storage/reduction catalysts: mechanism, materials and degradation studies. Catal. Sci. Tech..

[6] Dec JE (2009). Advanced compression-ignition engines—understanding the in-cylinder processes. Proc. Comb. Inst..

[7] Zhang Y, Cattrall RW, McKelvie ID, Kolev SD (2011). Gold, an alternative to platinum group metals in automobile catalytic converters. Gold. Bull..

[8] Herzing AA, Kiely CJ, Carley AF, Landon P, Hutchings GJ (2008). Identification of active gold nanoclusters on iron oxide supports for CO oxidation. Science.

[9] Carley AF (2011). CO bond cleavage on supported nano-gold during low temperature oxidation. Phys. Chem. Chem. Phys..

[10] Zhu Z (2013). Highly active and stable Co3O4/ZSM-5 catalyst for propane oxidation: effect of the preparation method. ACS Catal..

[11] Xie X, Li Y, Liu Z-Q, Haruta M, Shen W (2009). Low-temperature oxidation of CO catalysed by Co3O4 nanorods. Nature.

[12] Zhai Y (2010). Alkali-stabilized Pt-OHx species catalyze low-temperature water-gas shift reactions. Science.

[13] Ding K (2015). Identification of active sites in CO oxidation and water-gas shift over supported Pt catalysts. Science.

[14] Yang M (2015). A common single-site Pt(II)–O(OH)x– species stabilized by sodium on “Active” and “Inert” supports catalyzes the water-gas shift reaction. J. Am. Chem. Soc..

[15] Qiao B (2011). Single-atom catalysis of CO oxidation using Pt1/FeOx. Nat. Chem..

[16] Li X (2016). Single-atom Pt as co-catalyst for enhanced photocatalytic H2 evolution. Adv. Mater..

[17] Kyriakou G (2012). Isolated metal atom geometries as a strategy for selective heterogeneous hydrogenations. Science.

[18] Peterson EJ (2014). Low-temperature carbon monoxide oxidation catalysed by regenerable atomically dispersed palladium on alumina. Nat. Commun..

[19] Zhang Z (2017). Thermally stable single atom Pt/m-Al2O3 for selective hydrogenation and CO oxidation. Nat. Commun..

[20] Liu Jingyue (2016). Catalysis by Supported Single Metal Atoms. ACS Catalysis.

[21] Jones J (2016). Thermally stable single-atom platinum-on-ceria catalysts via atom trapping. Science.

[22] Wei S (2018). Direct observation of noble metal nanoparticles transforming to thermally stable single atoms. Nat. Nanotechnol..

[23] Nie L (2017). Activation of surface lattice oxygen in single-atom Pt/CeO2 for low-temperature CO oxidation. Science.

[24] USDRIVE. Aftertreatment Protocols For Catalyst Characterization and Performance Evaluation: Low Temperature Oxidation Catalyst Test Protocol. (2015).

[25] Curran SJ, Gao Z, Wagner RM (2015). Reactivity-controlled compression ignition drive cycle emissions and fuel economy estimations using vehicle system simulations. Int. J. Engine Res..

[26] Wang Y-G, Yoon Y, Glezakou V-A, Li J, Rousseau R (2013). The role of reducible oxide–metal cluster charge transfer in catalytic processes: new insights on the catalytic mechanism of CO oxidation on Au/TiO2 from ab initio molecular dynamics. J. Am. Chem. Soc..

[27] Yang Z (2015). Size-dependent CO and propylene oxidation activities of platinum nanoparticles on the monolithic Pt/TiO2-YOx diesel oxidation catalyst under simulative diesel exhaust conditions. Catal. Sci. Tech..

[28] Hoang Son, Lu Xingxu, Tang Wenxiang, Wang Sibo, Du Shoucheng, Nam Chang-Yong, Ding Yong, Vinluan Rodrigo D., Zheng Jie, Gao Pu-Xian (2019). High performance diesel oxidation catalysts using ultra-low Pt loading on titania nanowire array integrated cordierite honeycombs. Catalysis Today.

[29] Wu D, Zhang H (2013). Mechanical stability of monolithic catalysts: scattering of washcoat adhesion and failure mechanism of active material. Ind. Eng. Chem. Res..

[30] Yang X-F (2013). Single-atom catalysts: a new frontier in heterogeneous catalysis. Acc. Chem. Res..

[31] Baxter RJ, Hu P (2002). Insight into why the Langmuir–Hinshelwood mechanism is generally preferred. J. Chem. Phys..

[32] Newton MA, Ferri D, Smolentsev G, Marchionni V, Nachtegaal M (2015). Room-temperature carbon monoxide oxidation by oxygen over Pt/Al2O3 mediated by reactive platinum carbonates. Nat. Commun..

[33] Allian AD (2011). Chemisorption of CO and mechanism of CO oxidation on supported platinum nanoclusters. J. Am. Chem. Soc..

[34] Zammit, M. et al., Future Automotive Aftertreatment Solutions: The 150 °C Challenge Workshop Report. (National Technical Information Service, U.S. Department of Commerce 2012).

[35] Duprat F (2002). Light-off curve of catalytic reaction and kinetics. Chem. Eng. Sci..

[36] Heck RM, Farrauto RJ (2001). Automobile exhaust catalysts. Appl. Catal. A: Gen..

[37] Johnson J (2000). Cleaner vehicles, gas to be required by EPA. Chem. Eng. N..

[38] Kašpar J, Fornasiero P, Hickey N (2003). Automotive catalytic converters: current status and some perspectives. Catal. Today.

[39] Johannessen E, Wang G, Coppens M-O (2007). Optimal Distributor networks in porous catalyst pellets. I. Molecular diffusion. Ind. Eng. Chem. Res..

[40] Wang G, Coppens M-O (2008). Calculation of the optimal macropore size in nanoporous catalysts and its application to DeNOx catalysis. Ind. Eng. Chem. Res..

[41] Hoang S, Guo S, Hahn NT, Bard AJ, Mullins CB (2012). Visible light driven photoelectrochemical water oxidation on nitrogen-modified TiO2 nanowires. Nano Lett..

[42] Feng X (2008). Vertically aligned single crystal TiO2 nanowire arrays grown directly on transparent conducting oxide coated glass: synthesis details and applications. Nano Lett..

[43] Avila MS (2010). Effect of V2O5 loading on propane combustion over Pt/V2O5–Al2O3 Catalysts. Catal. Lett..

[44] Kresse G, Furthmüller J (1996). Efficiency of ab-initio Total energy calculations for metals and semiconductors using a plane-wave basis set. Comput. Mater. Sci..

[45] Kresse G, Furthmüller J (1996). Efficient iterative schemes for ab initio total-energy calculations using a plane-wave basis set. Phys. Rev. B.

[46] Perdew JP, Burke K, Ernzerhof M (1996). Generalized gradient approximation made simple. Phys. Rev. Lett..

[47] Grimme S (2006). Semiempirical GGA-type density functional constructed with a long-range dispersion correction. J. Comp. Chem.,.

[48] Henkelman G, Uberuaga BP, Jónsson H, Climbing Image A (2000). Nudged Elastic band method for finding saddle points and minimum energy paths. J. Chem. Phys..

