# Author Correction: Activating low-temperature diesel oxidation by single-atom Pt on TiO_2_ nanowire array

**DOI:** 10.1038/s41467-020-15227-7

**Published:** 2020-03-09

**Authors:** Son Hoang, Yanbing Guo, Andrew J. Binder, Wenxiang Tang, Sibo Wang, Jingyue (Jimmy) Liu, Huan Tran, Xingxu Lu, Yu Wang, Yong Ding, Eleni A. Kyriakidou, Ji Yang, Todd J. Toops, Thomas R. Pauly, Rampi Ramprasad, Pu-Xian Gao

**Affiliations:** 1 Department of Materials Science and Engineering & Institute of Materials Science, University of Connecticut, Storrs, CT 06269-3136 USA; 2 College of Chemistry, Central China Normal University, 430079 Wuhan, China; 3 Oak Ridge National Laboratory, Oak Ridge, TN 37932 USA; 4 Department of Physics, Arizona State University, Tempe, AZ 85287 USA; 5 Shanghai Synchrotron Radiation Facility, Shanghai Institute of Applied Physics, Chinese Academy of Sciences, 201800 Shanghai, China; 6 School of Materials Science and Engineering, Georgia Institute of Technology, Atlanta, GA 30332 USA; 7 Umicore Autocat USA Inc., Auburn Hills, MI 48326 USA

**Keywords:** Pollution remediation, Heterogeneous catalysis, Porous materials, Nanowires

Correction to: *Nature Communications* 10.1038/s41467-020-14816-w, published online 26 February 2020.

The original version of this Article contained an error in the spelling of the authors Huan Tran and Thomas R. Pauly, which were incorrectly given as Tran D. Huan and Thomas J. Pauly. This has now been corrected in both the PDF and HTML versions of the Article.

