# Supplementary material for: Activating low-temperature diesel oxidation by single-atom Pt on TiO2 nanowire array
Source: Nat Commun. 2020 Feb 26;11:1062. doi: 10.1038/s41467-020-14816-w (PMC7044320; doi:10.1038/s41467-020-14816-w)
Supplement: Supplementary file 1 — Supplementary Information [file 41467_2020_14816_MOESM1_ESM.pdf]

Supplementary Information for

Activating Low-temperature Diesel Oxidation by Single-Atom Pt on TiO_2 Nanowire Array

Son Hoang et al.

Supplementary Methods

Catalyst preparation

TiO_2 nanowire arrays are grown on ceramic monoliths via a nonpolar solvent/hydrophilic solid substrate interfacial reaction under hydrothermal conditions.[1, 2] Typically, the washed and TiO_2 seeded cordierite honeycomb substrates with size up to 7.5 cm × 7.5 cm × 5 cm are placed within a sealed Teflon reactor (1 L), containing 500 mL of a non-polar solvent, 50 mL of a Ti (IV) alkoxide precursor, 5 mL of titanium tetrachloride (1 M in toluene), and 50 mL of hydrochloric acid (37 wt.%). At room temperature, titanium (IV) precursors and water are separated since the Ti precursors are dissolved in the nonpolar solvent. Under hydrothermal conditions, water diffuses away from the high-energy water/nonpolar solvent interface to the hydrophilic TiO_2 nuclei on ceramic wall, where water reacts with Ti precursors, resulting in growth-crystallization of TiO_2. The presence of Cl^- anions is crucial for the anisotropic growth, as they tend to absorb on the rutile (110) plane, thus inhibiting further crystal growth of this plane. After being taken out for the reactor, all the samples are sonicated in acetone, ethanol, and water for 3 hours (h) to remove organic compounds and Cl^- residues from the synthesis before Pt loading.

Microwave-assisted dip-coating and Na-promoted wet incipient impregnation methods are employed to load Pt on the TiO_2 nano-arrays. For the dip-coating method, the substrate is first submerged into the diluted Pt precursor solution (0.5 mg Pt precursor per mL). It is taken out and blow-dried using compression air stream. The substrate is then dried in a microwave oven for 1-3 minutes. These steps are repeated until appropriate amount of the metal precursor is absorbed. Finally, the sample is calcined in air at 500 ^oC for 2-4 h with a ramp rate of 2 ^oC min^{-1}. In addition, a following 1-hour stay at a different calcination temperature such as 700 ^oC, 800 ^oC, and 900 ^oC has been adopted to look into the induced porosity and surface area change and its effect on the single atom loading and the catalyst stability. For impregnation, the substrate is submerged into diluted Pt precursor (50 $\mu g\ mL^{-1}$) and NaOH (atomic ratio Na/Pt is 10/1), and aged for 12 h at 80 ^oC. The solution is then evaporated in open air at 50 ^oC until dry. During evaporation, the substrate is rotated every 15-20 minutes to enhance uniformity. Finally, the sample is dried at 150 ^oC for 12 h and calcined in air at 500 ^oC for 4 h with a ramp rate of 2 ^oC min^{-1}.

To evaluate their hydrothermal stability in the probe reactions (CO and C_3H_6 oxidation) and the simulated exhaust (clean diesel combustion, CDC; and low temperature combustion-diesel, LTC-D) tests, the monolithic catalysts are aged in a flow of 12 % O_2, 6 % CO_2, 6 % H_2O in N_2 at 700 ^oC for 100 h. In the transient engine tests, the sample is aged in a flow on 10 % H_2O in air at 650 ^oC for 100 h. These aging conditions are chosen to represent a North America heavy-duty diesel application.

Catalytic activity evaluation

The catalytic activity measurements were performed in a continuous flow reactor equipped with a gas chromatography. The monolithic catalysts (5 × 5 channels × 2.5 cm long) were placed in a tubular quartz reactor with a surrounding electrical heating coil. The inlet temperature was measured using a 0.16 mm K-type thermocouple, which was fixed at < 5 mm in front of the monolith. The utilization of the small thermocouple (0.16 mm) is to prevent disruption of gas flow. Typically, the catalytic activity of each sample was evaluated through total 6 reaction cycles, in which the inlet temperature was ramped up from 100 ^oC to 525 ^oC at 2 ^oC min^{-1} and naturally cool down to 100 ^oC before next testing cycles. The feed gas mixtures were controlled by mass flow controller before entering the blender and then the reactor at a gas hourly space velocity of 30,000 h^{-1}. The feed gas always contains 12% O_2 to mimic the O_2 concentration in the CDC and LTC-D simulated exhaust, but varies CO and C_3H_6 concentrations (also were chosen to reflect the CO and total hydrocarbon concentration in the CDC and LTC-D simulated exhausts) in each cycle such that: cycle 1 and 2 contain 2000 ppm CO and 1000 ppm C_3H_6; cycle 3 contains 2000 ppm CO; cycles 4 contains 1000 ppm C_3H_6; cycle 5 contains 500 ppm C_3H_6; and cycle 6 contains 500 ppm CO and 500 ppm C_3H_6; all are balanced by N_2. Sample Array-50-D was evaluated in an additional sequence (cycle 3→4→5→6→1→2), showing negligible difference in the catalytic activity, thus confirming the sequence of the tests does not affect the catalytic activity of TiO_2 nano-array supported Pt catalysts.

The catalytic diesel oxidation activity of catalysts under simulated exhaust conditions was evaluated according to the protocol developed by the Advanced Combustion and Emission Control (ACEC) Technical Team of USDRIVE. The light-off measurements were conducted on a customized plug-flow reactor system. Micro-cores of catalyst samples were cut and loaded into cylindrical quartz tubing using a wrapping in order to ensure no gas could bypass the catalyst channels. The catalyst temperature was monitored using two thermocouples, one measuring the inlet temperature while located ~2 cm from the catalyst front, the other measuring the mid-catalyst temperature and located from the rear into the middle of the central channel of the micro-core. Space velocity was kept at 60,000 h^{-1} throughout all the tests. Gas concentrations were determined per ACEC Tech Team (USDRIVE) protocol. The composition of the "LTC-D" simulated exhaust is $[O_2]$ = 12%, $[H_2O]$ = 6%, $[CO_2]$ = 6%, $[H_2]$ = 400 ppm, [CO] = 2000 ppm, [NO] = 100 ppm, $[C_2H_4]$ = 833.5 ppm, $[C_3H_6]$ = 333 ppm, $[C_3H_8]$ = 111 ppm, and Ar balance. The composition of the "CDC" simulated exhaust is $[O_2]$ = 12%, $[H_2O]$ = 6%, $[CO_2]$ = 6%, $[H_2]$ = 100 ppm, [CO] = 500 ppm, [NO] = 200 ppm, $[C_2H_4]$ = 389 ppm, $[C_3H_6]$ = 233.5 ppm, $[C_3H_8]$ = 51.7 ppm, and Ar balance. Water was introduced via argon flow through a bubbler and heated lines held at 200 °C. Product gas stream was measured via MKS FTIR. Experiments were conducted first with a degreening step in only the O_2, CO_2, and H_2O components of the gas stream at 700 °C for 4 h for LTC-D and 550 ^oC for 4 h CDC protocols, followed by an evaluation step from 100 °C to 500 °C at a rate of 2 °C min^{-1}. Besides the regular simulated exhaust test, catalyst samples were also treated using the following simulated exhaust test treatment protocol to look into the single Pt atom species fate before and after experiencing the treatment on the Pt/TiO_2 NA samples with both fresh state and hydrothermal aged condition. CDC simulated gas treatment: 12% O_2 + 6% H_2O + 6% CO_2 +100ppm H_2 + 500ppm CO + 200ppm NO +

1400ppm HCs, with a ramp rate of 5 ^oC min^{-1} from room temperature to 500 ^oC, and then keeping at 500 ^oC for 1h.

Sulfur poisoning effects on the catalyst performance were evaluated following USDRIVE's protocol. The catalyst was exposed to 5 ppm SO_2 added to the full simulated exhaust at a space velocity of 30,000 h^{-1} and 300 ^oC catalyst inlet temperature for 5 h. A total sulfur exposure level of 1 g sulfur per liter of catalyst was estimated following this exposure condition. Poisoning was conducted following the pretreatment in which the catalyst was annealed at 600 ^oC for 20 minutes before cooled down to 300 ^oC in 12 % O_2, 6% H_2O, 6 % CO_2, and N_2 balance. After sulfur exposure, SO_2 was removed from the feed and the sample cooled to 100 °C at which point the catalyst performance was evaluated. For de-sulfation, the catalyst was annealed at 700 ^oC in 3% H_2 and 1% CO in N_2 for 30 mins, followed by calcination in air at 500 ^oC for 2 h.

The Pt/TiO_2 nano-array sample with a Pt loading of 0.53 g L^{-1} and dimensions of Φ 2" × 3" were tested under highly transient feed gas conditions mimicking a heavy-duty diesel (HDD) federal test procedure (FTP) as running on a HDD certified 2010 Cummins ISB (6.7 L) 320 hp engine. The characteristic of the transient feed gas is provided in Supplementary Fig. 19. The Pt/TiO_2 nano-array and reference samples were aged at 650 ^oC for 100 h in 10% steam/air flow to a representative end of life condition for a North American HDD application.

Catalyst characterization

The X-ray absorption fine structure data was collected at the BL14W1 station at the Shanghai Synchrotron Radiation Facility (SSRF). The storage ring of SSRF was operated at 3.5 G eV with a maximum current of 260 mA. Pt foil and PtO_2 were used as reference samples. Using Si (111) double-crystal monochromator, the data collection was carried out in fluorescence mode for Pt L_3-edge with a 7-element detector Ge solid state detector. The energy was calibrated according to the absorption edge of Pt foil (edge energy: 11564eV).

The EXAFS data was analyzed and fitted using the ATHENA module in the IFEFFIT software packages. Each k^2-weighted EXAFS spectrum was processed by a two-step procedure: i) subtraction of post-edge background from overall absorption; and ii) normalization with respect to the edge-jump step. The k^2-weighted χ(k) data of Pt L_3-edge were then Fourier transformed to real (R) space through a hanning windows (dk=1.0 $Å^{-1}$) in order to separate the EXAFS contributions from different coordination shells. To determine the quantitative structural parameters around central atoms, the ARTEMIS module of IFEFFIT software packages was employed for least-squares curve parameter fitting.

Coordination number of model samples (Pt foil) was fixed as the nominal value. The obtained S_0^2 of Pt foil was 0.80. It was fixed in the subsequent fitting of Pt L_3-edge date. The fitted ranges for k and R spaces were selected to be k =3–10.3 $Å^{-1}$ (k^2 weighted) with R=1.0–2.2 Å (Pt_1/TiO_2 NW) or 1.4–3.2 Å (Pt NP/TiO_2 NA).

To conduct the H_2 temperature programmed reduction (TPR), the samples were degassed in helium at ambient temperature for 20 minutes. TPR condition: 10% H_2, balance Ar, 50 ml min^{-1}, 10 °C min^{-1}. To determine the surface area and pore size distribution of the catalysts, N_2 adsorption–desorption isotherms and H_2 chemisorption were carried out using a Micromeritics ASAP 2020 Physisorption analyzer at liquid nitrogen temperature. Brunauer–Emmett– Teller (BET) method and Barret–Joyner–Halenda (BJH) method were used to calculate the specific surface area and pore size, respectively. . The samples to be measured were degassed at 350 ^oC for 4 h in vacuum before the measurement of N_2 adsorption-desorption isotherm.

The morphology and structure of catalysts were characterized by electron microscopies. Scanning electron microscopy images were taken using FEI Teneo low vacuum SEM and a JEOL 6335F field emission SEM, operating at 10-20 kV. Transmission electron microscopy (TEM) including both bright field and high angular annual dark field (HAADF) images and energy-dispersive X-ray spectroscopy for composition distribution were taken using a FEI Talos STEM and a Tecnai F30 STEM. The aberration corrected HAADF STEM images were obtained on two STEM/TEM systems: 1) a JEOL JEM 2200FS, attached with a CEOS (Heidelburg, Germany) probe corrector, with a nominal image resolution of 0.07 nm; and 2) a JEOL JEM-ARM200F, equipped with a CEOS probe corrector, with a guaranteed resolution of 0.08 nm. The chemical states of the TiO_2 and Pt/TiO_2 nanowire samples were characterized using a Thermo K-Alpha X-ray photoelectron Spectrometer with an Aluminum K-Alpha 1.486 KeV source. X-ray diffraction (XRD) patterns of the samples were collected by a Bruker D2 Phaser using Cu Kα radiation (λ = 0.15418 nm) operated at 40 kV and 40 mA. The XRD data were recorded for 2θ values from 10° to 80° with an interval of 0.02°.

H_2 chemisorption was used to determine the platinum dispersion (D_{Pt}) at 35 ^oC on a Micromeritics ASAP 2020 analyzer. H_2 reduction treatment of samples was conducted at 300 ^oC for 2 h, followed by the outgassing at 150 ^oC for 2 h prior to chemisorption experiment. A double isotherm method was used to determine the H_2 uptake: first isotherm revealed the total hydrogen consumed (uptake), $(HC)_T$, while the second one, acquired after evacuation for 2 h showed the ‘‘reversible’’ or weakly adsorbed hydrogen, (HC)r. The amount of ‘‘irreversible’’ or strongly adsorbed hydrogen, (HC)i, was determined by calculating the isotherm difference. The pressure range of isotherms was controlled at 0–12 kPa, where the extrapolation to zero pressure was used to measure gas uptake. The D_{Pt} was finally calculated by an atomic (HC)i/Pts = 1 ratio, where Pts implies a Pt atom on surface [3].

To evaluate the mechanical stability of TiO_2 nano-array integrated cordierite honeycomb samples, a sonication experiment was conducted on both TiO_2 and Pt/TiO_2 nano-array samples before and after hydrothermal aging at 700 ^oC for 100 h. The samples were sonicated in water at 40 kHz for 3 h and weighed before and after the sonication. Little morphology change (in SEM) was observed and the weight loss after the sonication process is less than 1 wt. % for both fresh and aged samples, indicating the sound

honeycomb structure integrity and the superior adhesion of TiO_2 and TiO_2/Pt nano-arrays on the honeycomb monolith channel walls.

Density Functional Theory (DFT) calculations were performed as implemented in the Vienna Ab initio simulation package [4, 5], employing the Perdew, Burke and Ernzerhof exchange-correlation functional [6]. Models for rutile TiO_2 (110) surfaces contain two layers along the y direction followed by a vacuum slab of more than 20 Angstrom. The (110) surface was constructed by repeating the cell of dimension 13.16Å × 11.88Å along the x and z directions, respectively. With this model, a k-point mesh of 2×1×2 and a plane-wave energy cutoff of 400 eV were used. As systems to be modeled involve isolated molecules like CO_2 and O_2, the long-range van der Waals interactions was included into the calculations using the scheme of Grimme [7]. Reaction pathways were searched using the nudge-elastic band method by Henkelman and Jónsson [8].

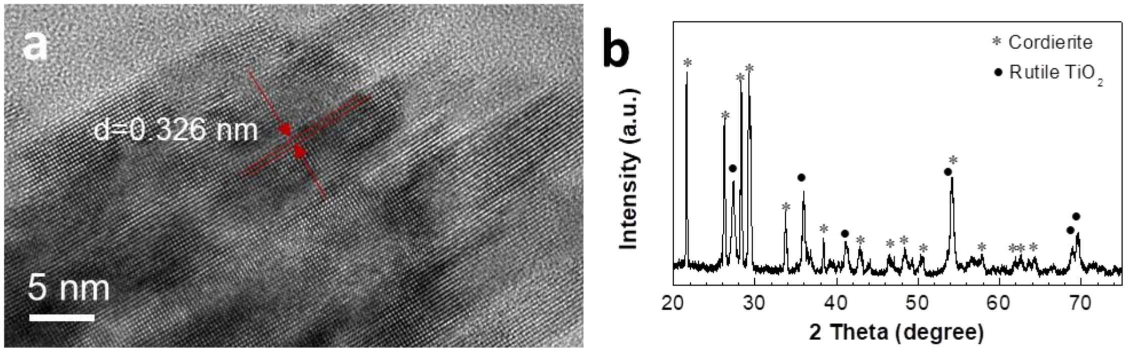

Supplementary Fig. 1 (**a**) TEM and (**b**) XRD of TiO_2 nano-array grown on cordierite monolith. The d-spacing of 0.326 nm identified from the TEM image corresponds to the d-spacing of {110} facets of rutile TiO_2.

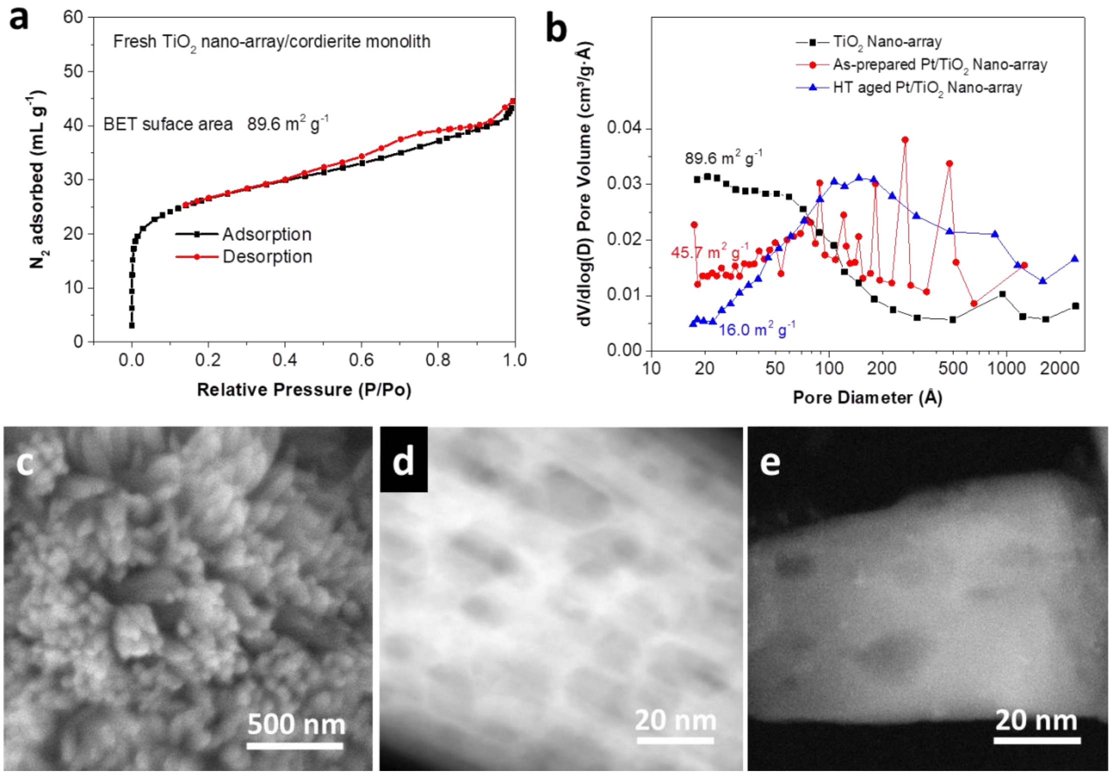

Supplementary Fig. 2 (**a**) N_2 isothermal adsorption-desorption as-prepared TiO_2 nano-array/cordierite substrates using butanone as solvent. (**b**) the BJH pore-size distribution of TiO_2 nano-array: as-prepared, after Pt loading (including calcination), and after hydrothermal aging (**c**) SEM image of hydrothermal aged Pt/TiO_2 nano-array indicating well-retained nano-array structure (**d, e**) HAADF of Pt/TiO_2 nano-array (**d**) before and (**e**) after hydrothermal aging indicating the closure of some mesopores (dark spots). Note: sample was hydrothermal aged at 700 ^oC for 100 h in 12 % O_2, 6 % H_2O, 6 % CO_2, and balance N_2.

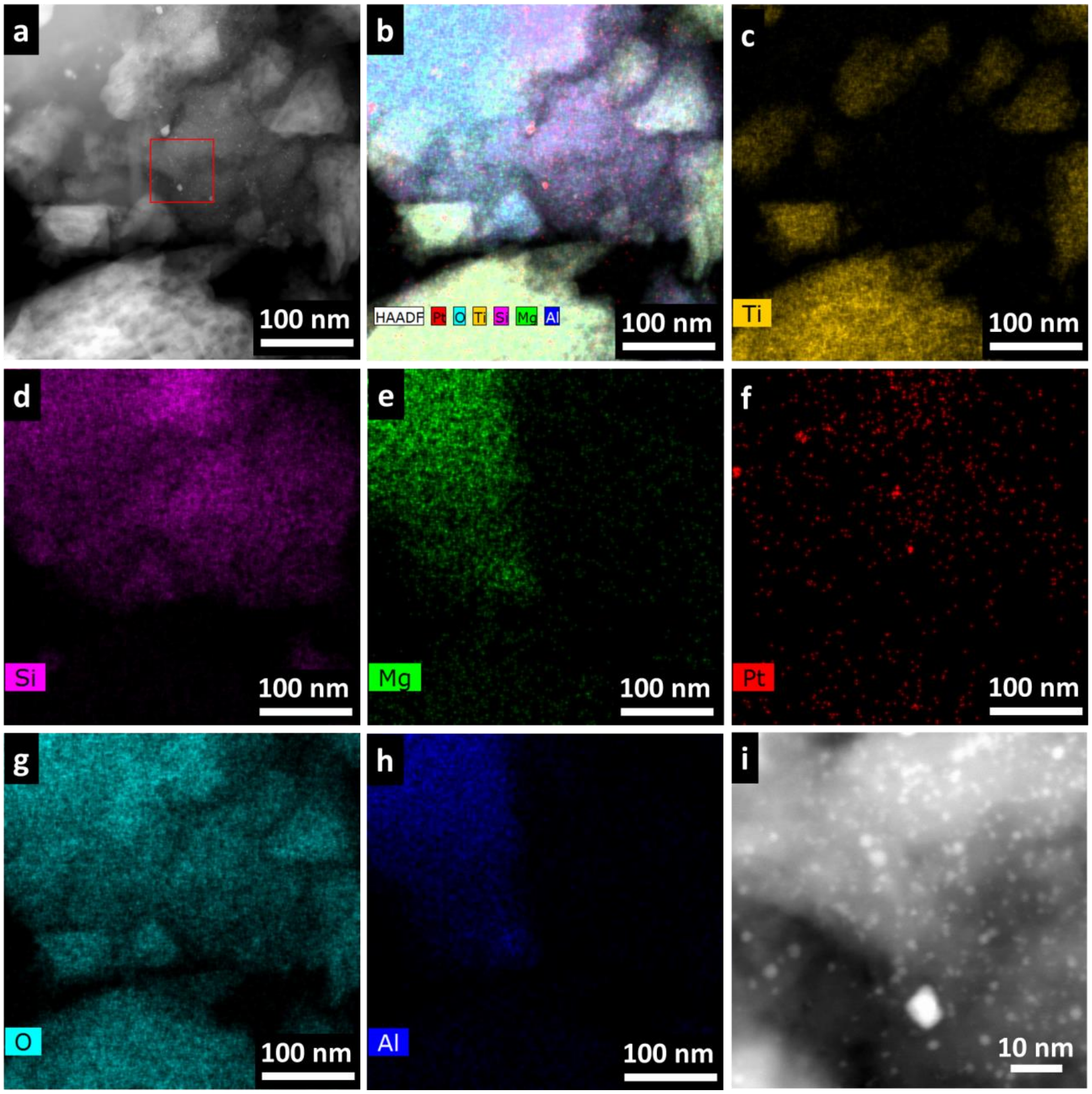

Supplementary Fig. 3 HAADF STEM image of Pt/TiO_2/SiO_2/cordierite sample (**a**) and the corresponding elemental mappings (**b-h**). (**i**) Magnified view of the Pt/SiO_2 area - the area in the red box in panel A. The SiO_2 was formed via acidic etching Fe, Mg, and Al from cordierite (a magnesium iron aluminum cyclosilicate). We noticed that Pt nanoparticles were mainly deposited on the SiO_2 area. Very few-to-none Pt nanoparticles are visible in the TiO_2 area.

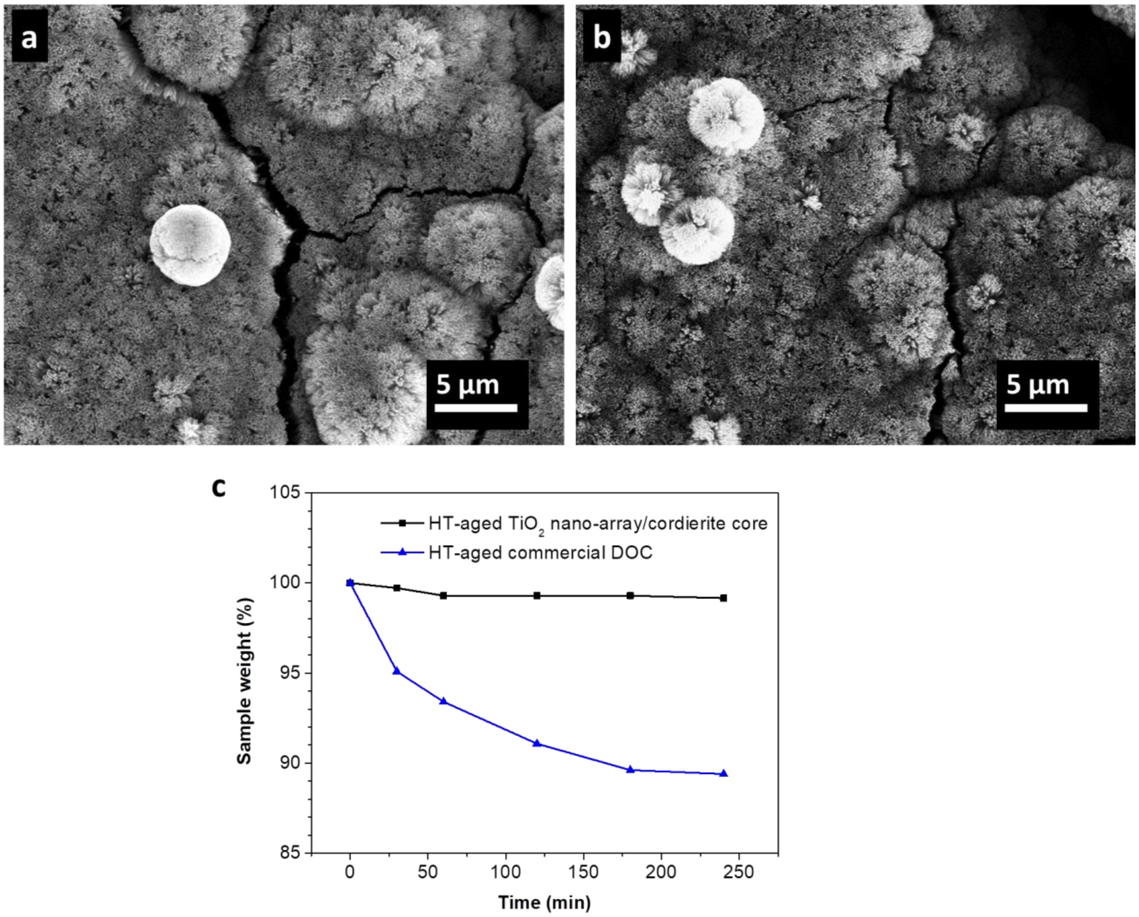

Supplementary Fig. 4 Mechanical stability evaluation of TiO_2 nano-array/cordierite monolith. Top-view SEM images of TiO_2 nano-array/cordierite monolith before (**a**) and after (**b**) 3-hour sonication in a water bath at 44 kHz, showing negligible morphology changes of TiO_2 nano-array due to ultrasonic vibration. (**c**) Weight-loss over time of hydrothermally aged TiO_2 nano-array and commercial DOC samples.

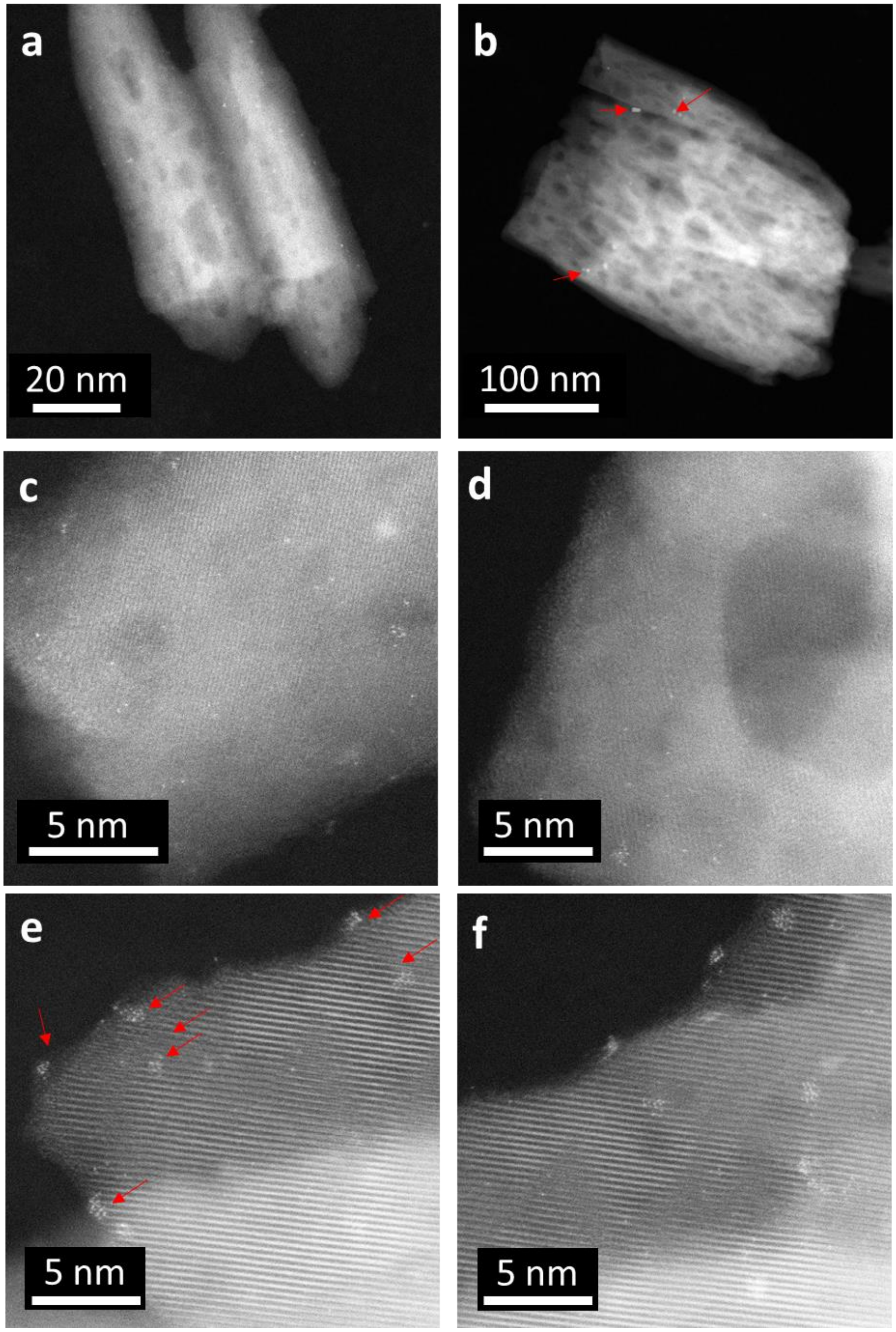

Supplementary Fig. 5 ac-HAADF STEM images of Pt/TiO_2 nano-array prepared by Na-promoted wet impregnation. Few Pt nanoparticles were occasionally found (**a, b**), the majority Pt was distributed as single atoms (**c, d**) and several sub-nm clusters (**e, f**).

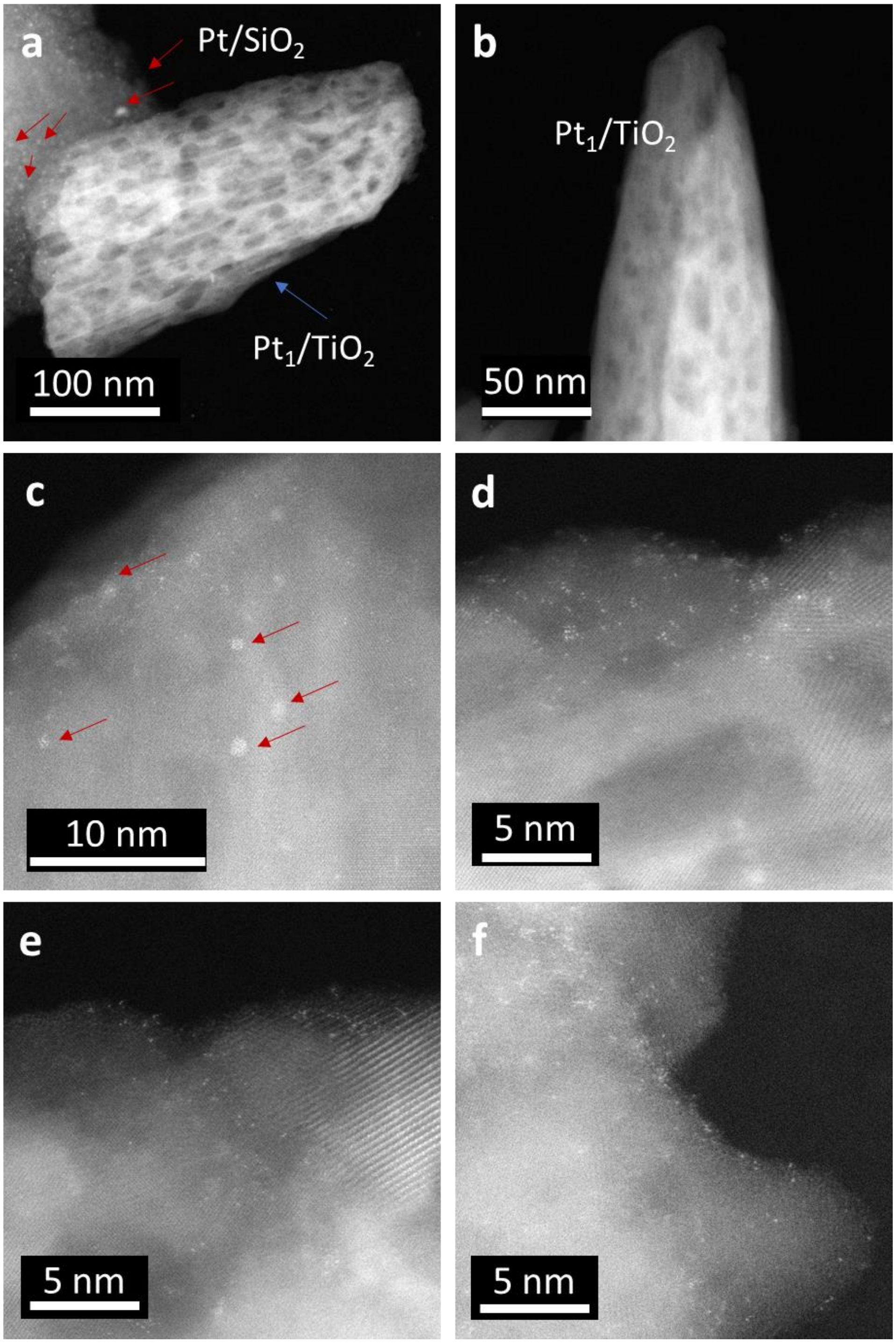

Supplementary Fig. 6 ac-HAADF STEM images of Pt/TiO_2 nano-array prepared by microwave-assisted dip coating. Pt nanoparticles were mainly deposited on the adjacent SiO_2 area while very few were visible on TiO_2 (**a, b**). On TiO_2, Pt was dominantly distributed single atoms (**d-f**) and several subnanometer clusters (**c**).

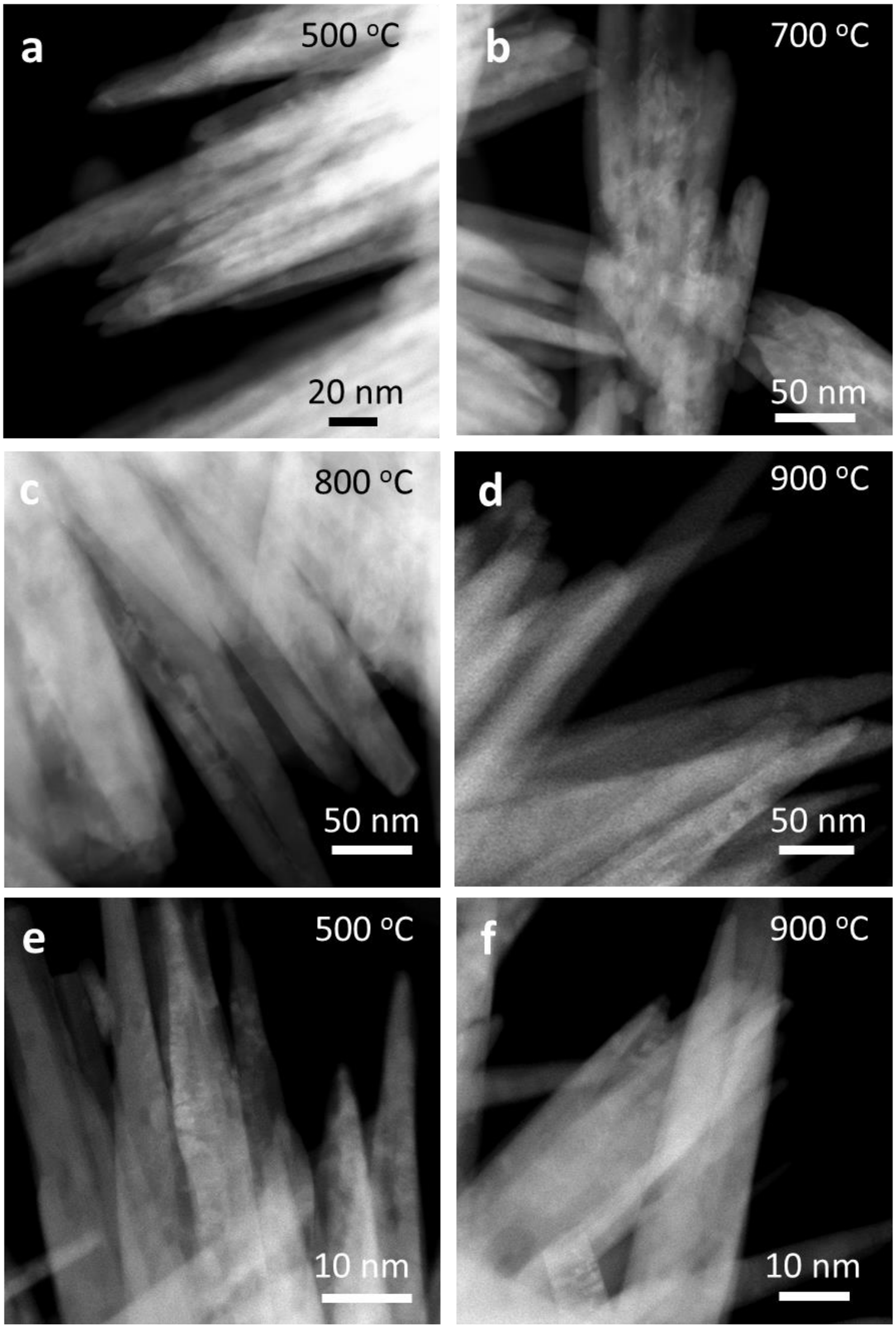

Supplementary Fig. 7 HAADF-STEM images of Pt_1/TiO_2 NA calcined at different temperatures (**a**) 500 ^oC, (**b**) 700 ^oC, (**c**) 800 ^oC, and (**d**) 900 ^oC; and Pt_1/TiO_2 NA calcined at 500 ^oC (**e**) and 900 ^oC (**f**) followed by 700 ^oC $\times$ 100 h hydrothermal aging and the CDC simulated exhaust test treatment. Few-to-None Pt nanoparticles were observed in these nanowire samples.

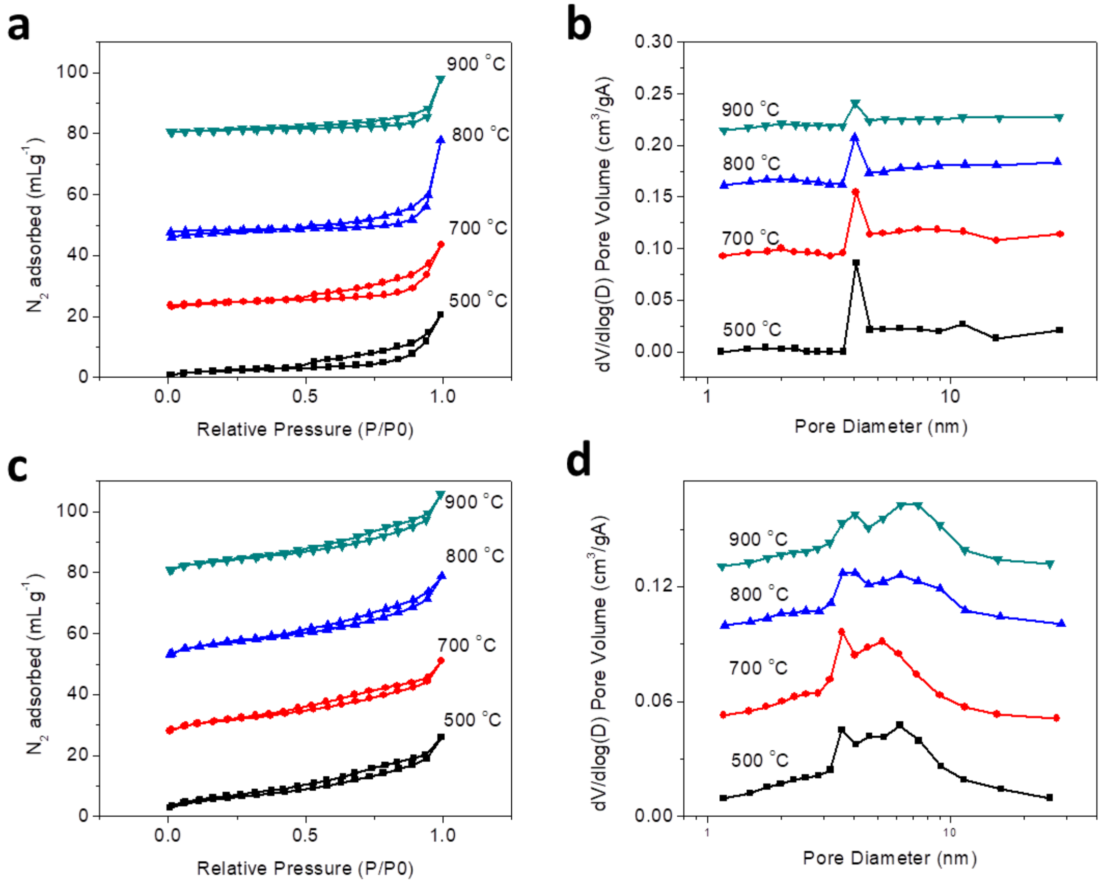

Supplementary Fig. 8 N_2 isothermal adsorption-desorption and the corresponding BJH pore-size distribution of (**a, b**) Pt/TiO_2 nanowire powders, and (**c, d**) Pt/TiO_2 nano-arrays prepared with different calcination temperatures.

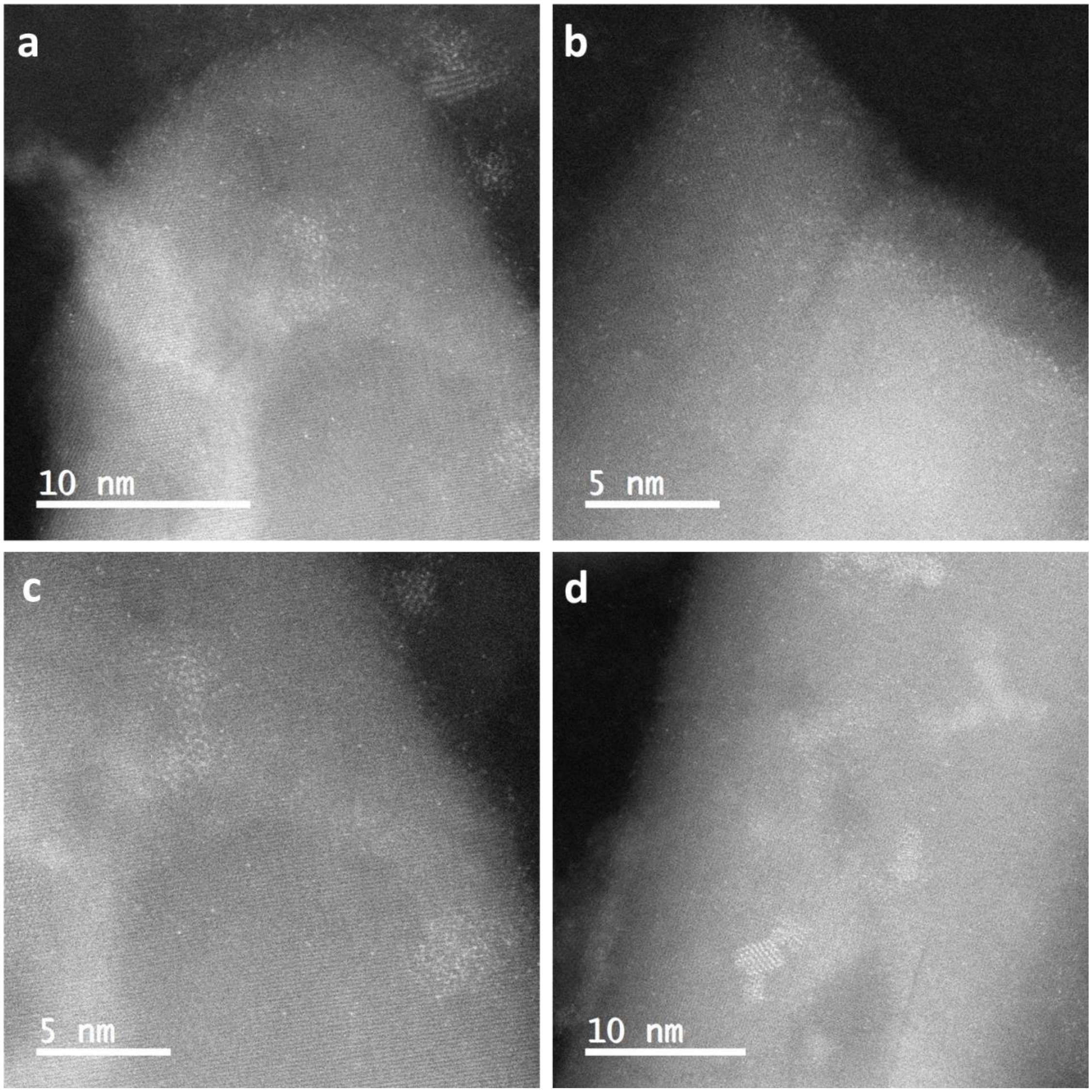

Supplementary Fig. 9 ac-HAADF STEM images of Pt/TiO_2 nano-array (prepared by microwave-assisted dip coating) after hydrothermal aging at 700 ^{o}C for 100 h. Pt was dominantly distributed as single atoms and raft-like clusters (**a-d**).

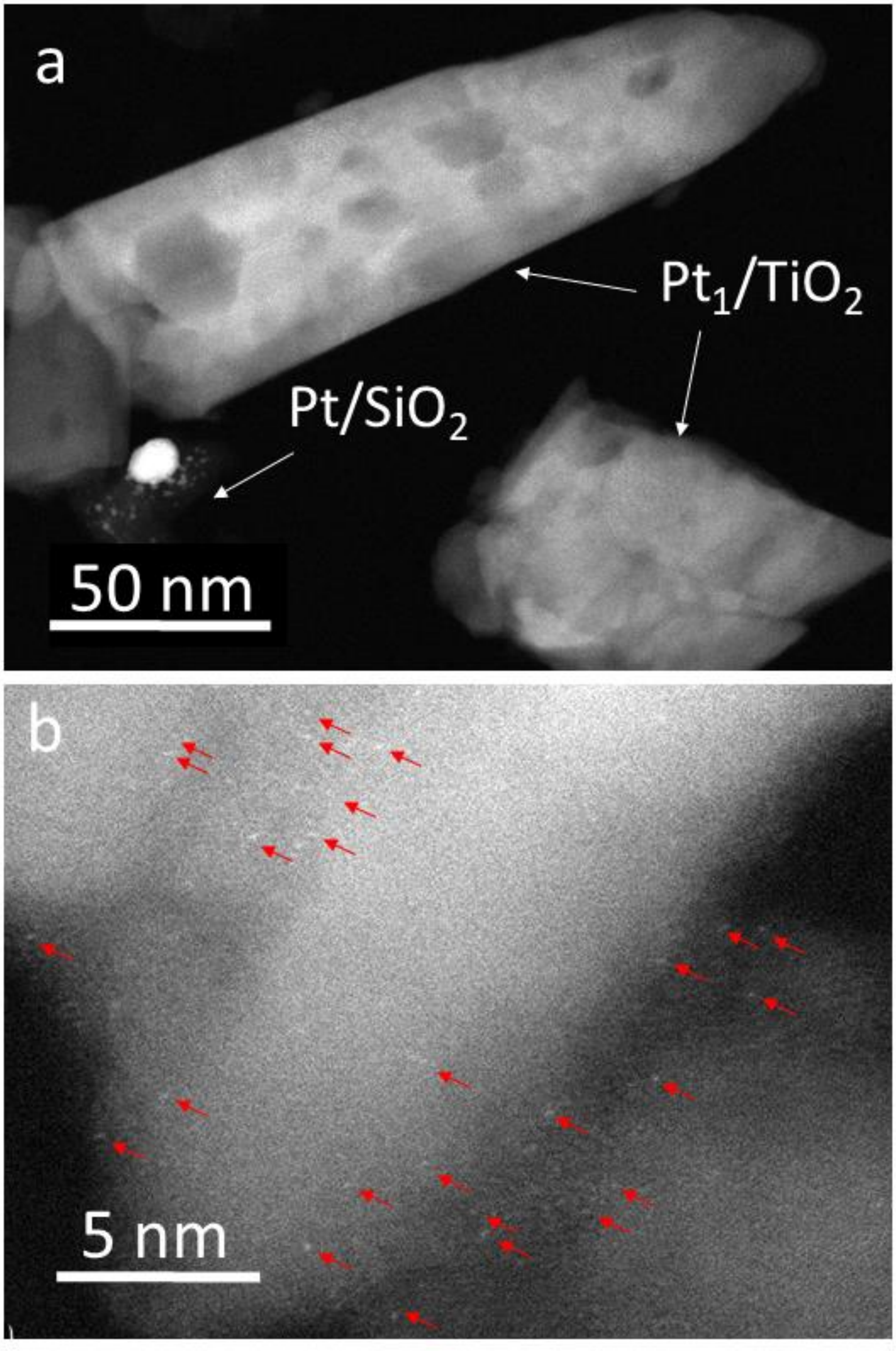

Supplementary Fig. 10 ac-HAADF STEM images impregnation Pt_1/TiO_2 nano-array after hydrothermal degreening at 700 ^oC for 4 h. Pt single atom dispersion is revealed on TiO_2 (**a-b**), while Pt is distributed as nanoparticles on SiO_2 (**a**) and sintered after degreening.

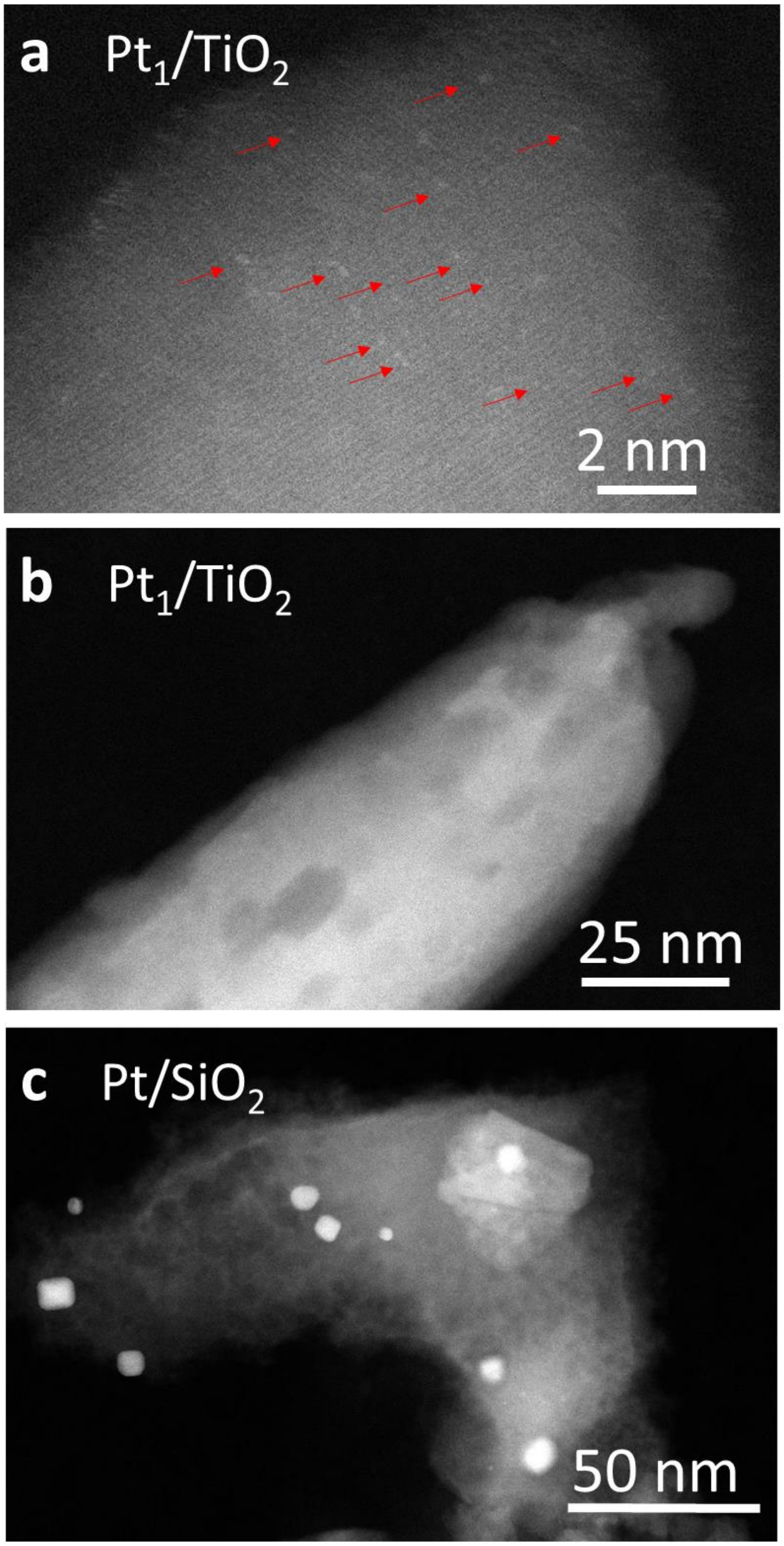

Supplementary Fig. 11 ac-HAADF STEM images of dip-coating Pt_1/TiO_2 nano-array hydrothermal degreening at 700 °C for 4 h. Pt single atom dispersion is revealed on TiO_2 (**a-b**), while on the adjacent SiO_2, Pt is distributed as big nanoparticles (**c**).

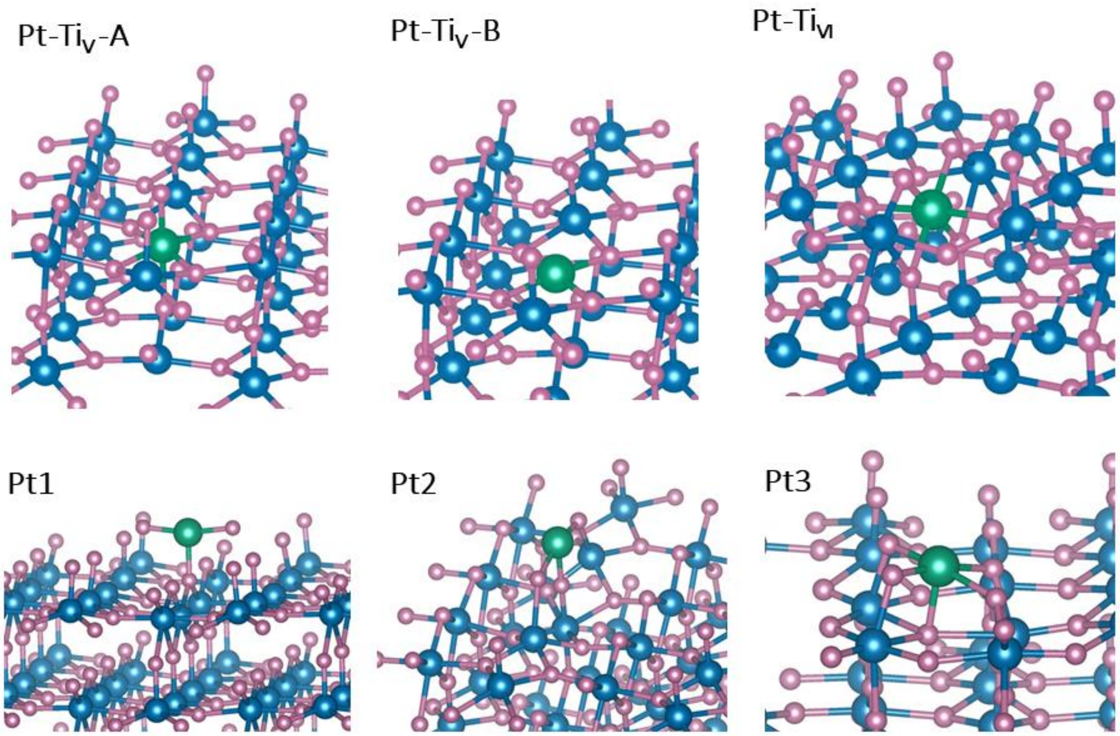

Configuration	Pt-Ti$_V$-A	Pt-Ti$_V$-B	Pt-Ti$_{VI}$	Pt1	Pt2	Pt3
**Pt binding energy (eV)**	10.44	11.09	8.78	4.25	6.26	9.27

Supplementary Fig. 12 Different configurations of isolated Pt atom supported on rutile TiO_2 (110) and their corresponding binding energies. The binding energy is defined as the energy needed to remove Pt atom from the surface to vacuum (distance of at least 10 Å from the surface).

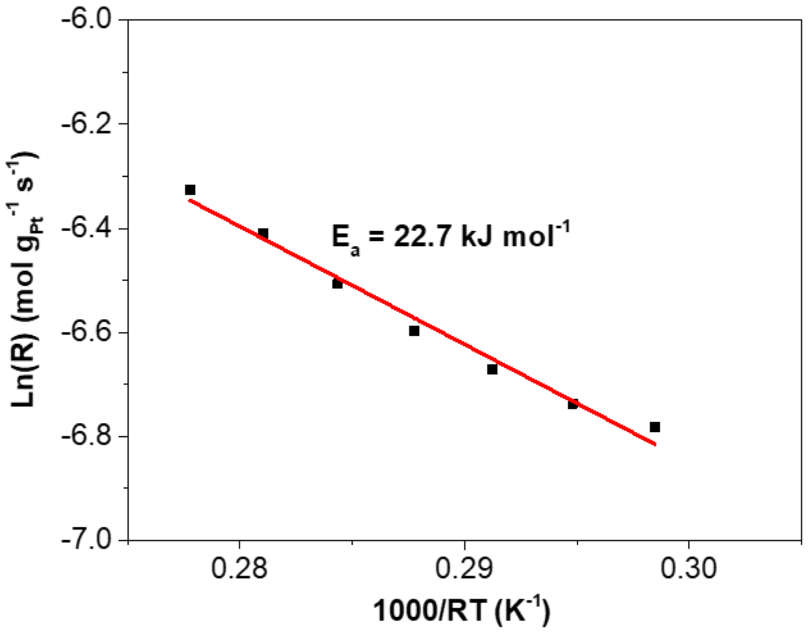

Supplementary Fig. 13 Arrhenius plot for CO oxidation on 0.2 wt.% Pt_1/TiO_2 NR. The feed gas is 2000 ppm CO, 12% O_2, and N_2 balance to reflect the CO concentration in the LTC-D simulated exhaust.

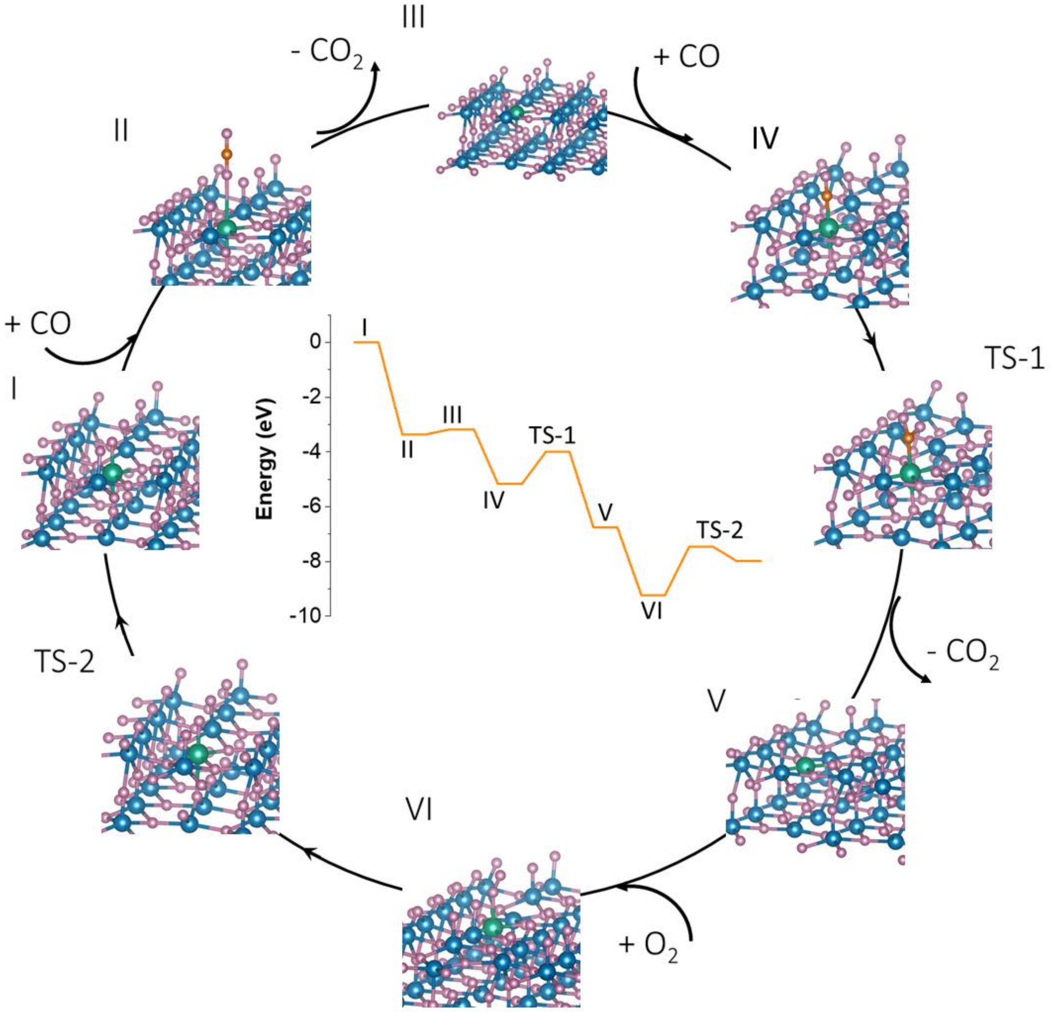

Supplementary Fig. 14 LH mechanism for CO oxidation over Pt_1/TiO_2 NA. The reaction cycle shows the structure of intermediates and transient state (TS) of the key elementary steps. The inset shows the calculated energy profile. The required activation energy is 1.8 eV for the dissociation of the adsorbed O_2 molecule.

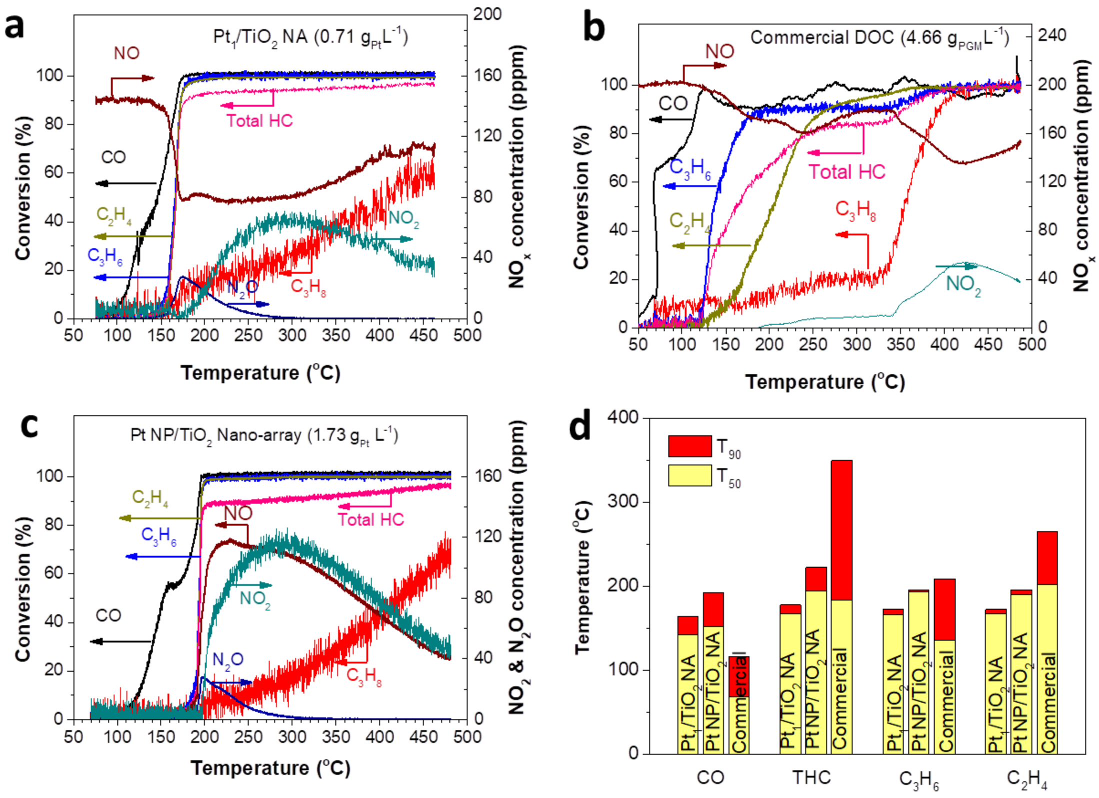

Supplementary Fig. 15 (**a-c**) Light-off curves in the CDC simulated exhaust for (**a**) Pt_1/TiO_2 NA, (**b**) Commercial DOC, and (**c**) Pt NP /TiO_2 NA. (**d**) Comparison of the activities of these samples.

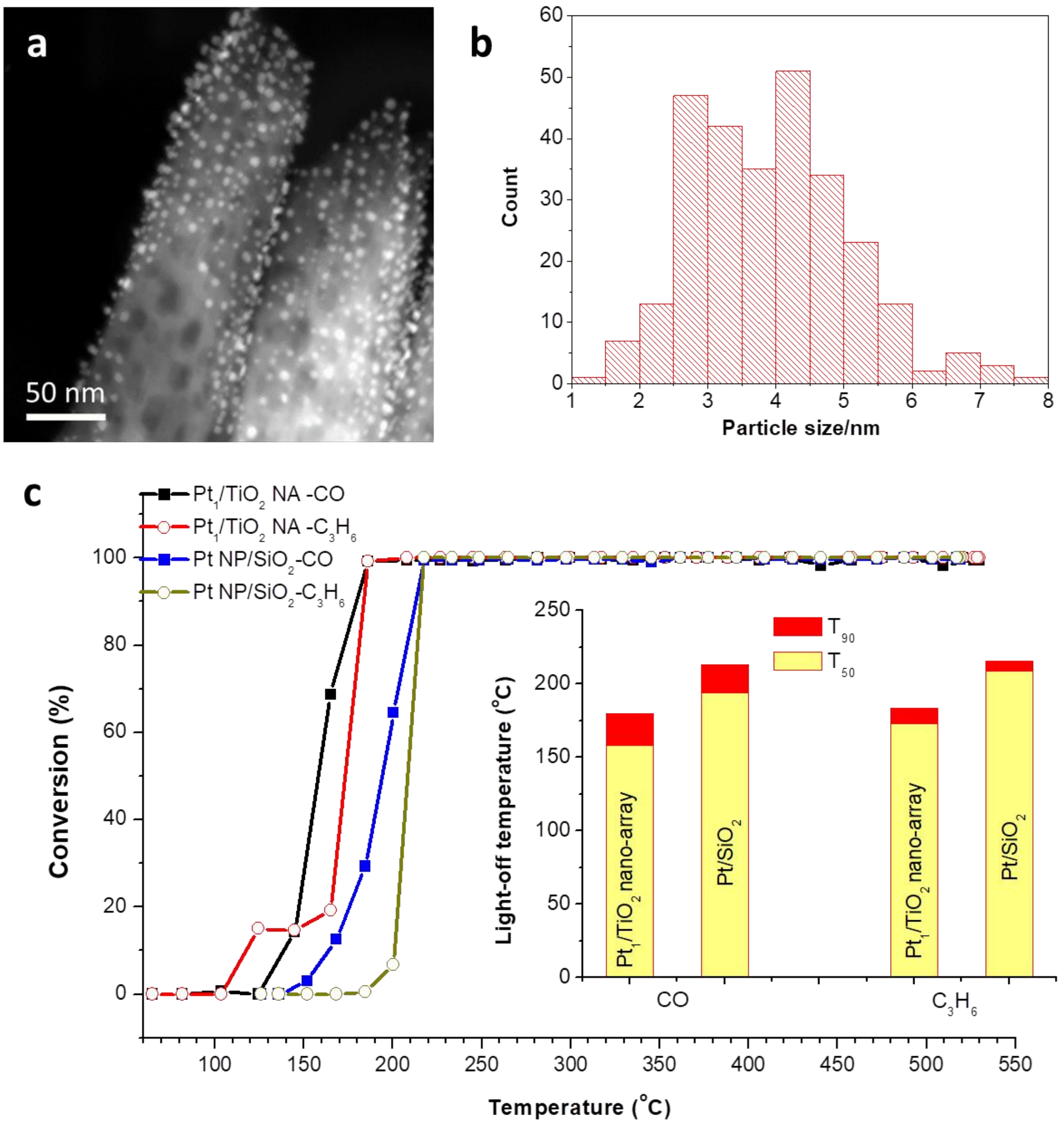

Supplementary Fig. 16 (**a**) HAADF TEM image and (**b**) the corresponding particle size histogram of Pt nanoparticles/TiO_2 nano-array monolith, prepared via atomic layer deposition of Pt over TiO_2 nano-array monolith. (**c**) Catalytic CO and C_3H_6 oxidation performance over Pt_1/TiO_2 NA and Pt/SiO_2 on etched cordierite monoliths with a Pt loading of 0.71 $g_{Pt}L^{-1}$.

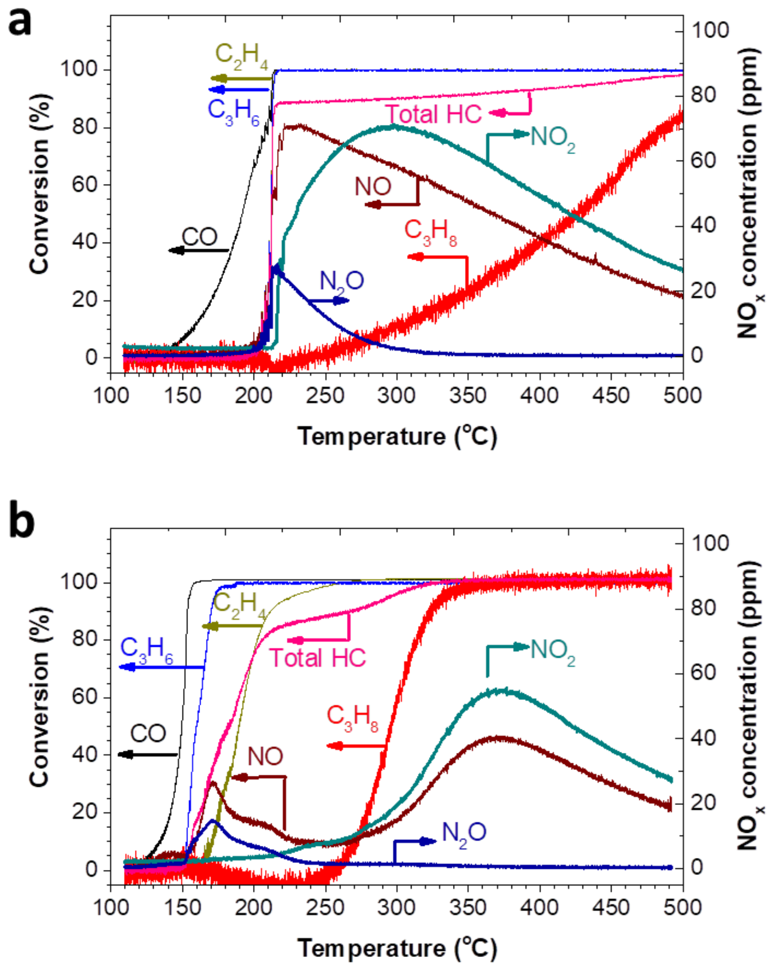

Supplementary Fig. 17 Light-off curves in the LTC-D simulated exhausts for (**a**) Pt_1/TiO_2 NA (1.73 $g_{Pt}L^{-1}$) and (**b**) Commercial DOC (4.66 g_{PGM} L^{-1})

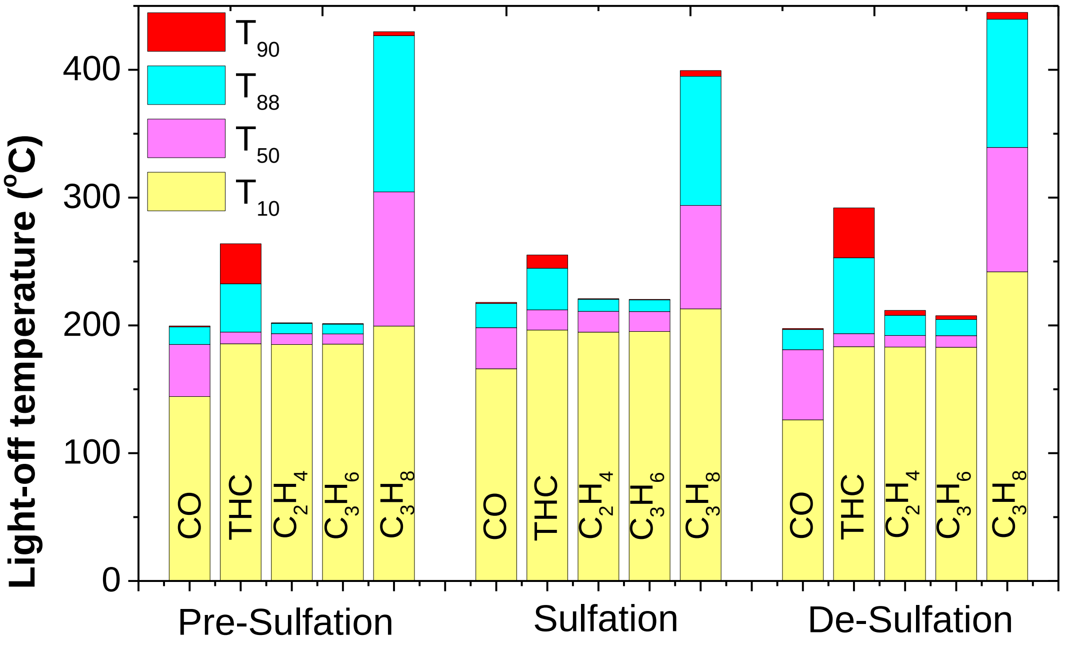

Supplementary Fig. 18 S-poisoning effect on the DOC activity of Pt_1/TiO_2 NA (1.73 g_{Pt} L^{-1}) in the LTC-D simulated exhaust.

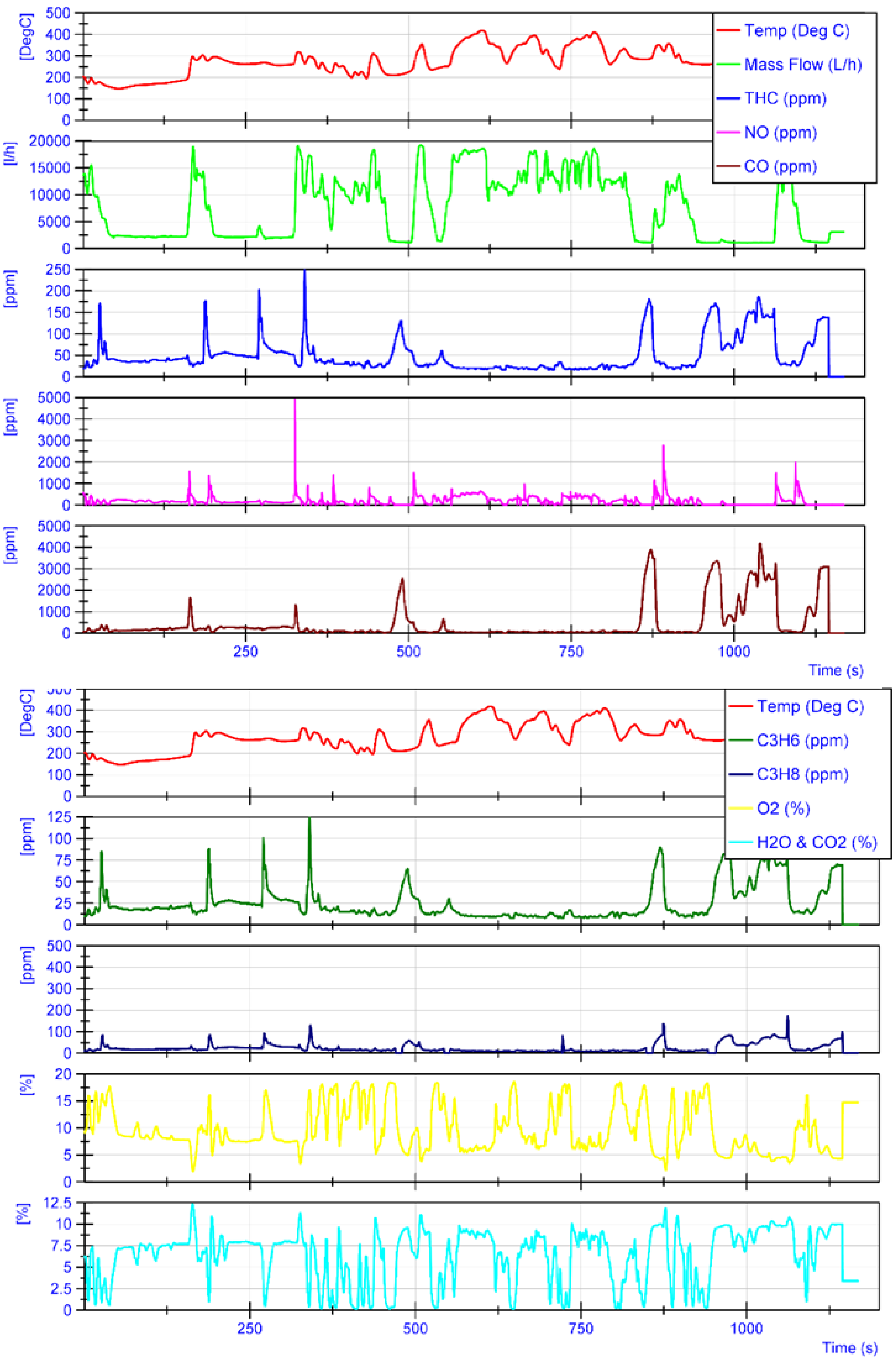

Supplementary Fig. 19 The characteristics of the transient feed gas.

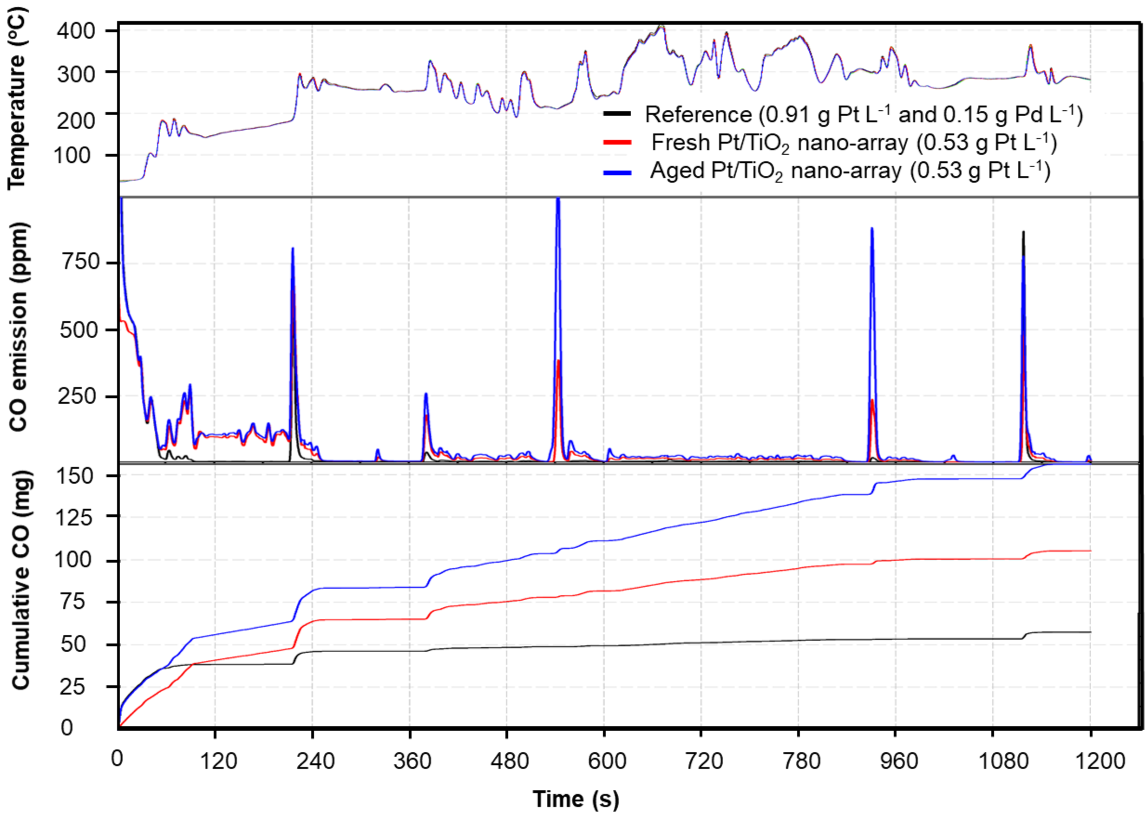

Supplementary Fig. 20 DOC activity of Pt_1/TiO_2 nano-array (0.53 g Pt L^{-1}) in transient gas conditions mimicking a HDD FTP as running on a HDD certified 2010 Cummins ISB (6.7 L) 320 hp engine. From top to bottom: temperature profile, transient CO emission, and cumulative CO emission. A commercial DOC (PGM loading: 0.91 g Pt/0.15 g Pd L^{-1}) was employed as a reference.

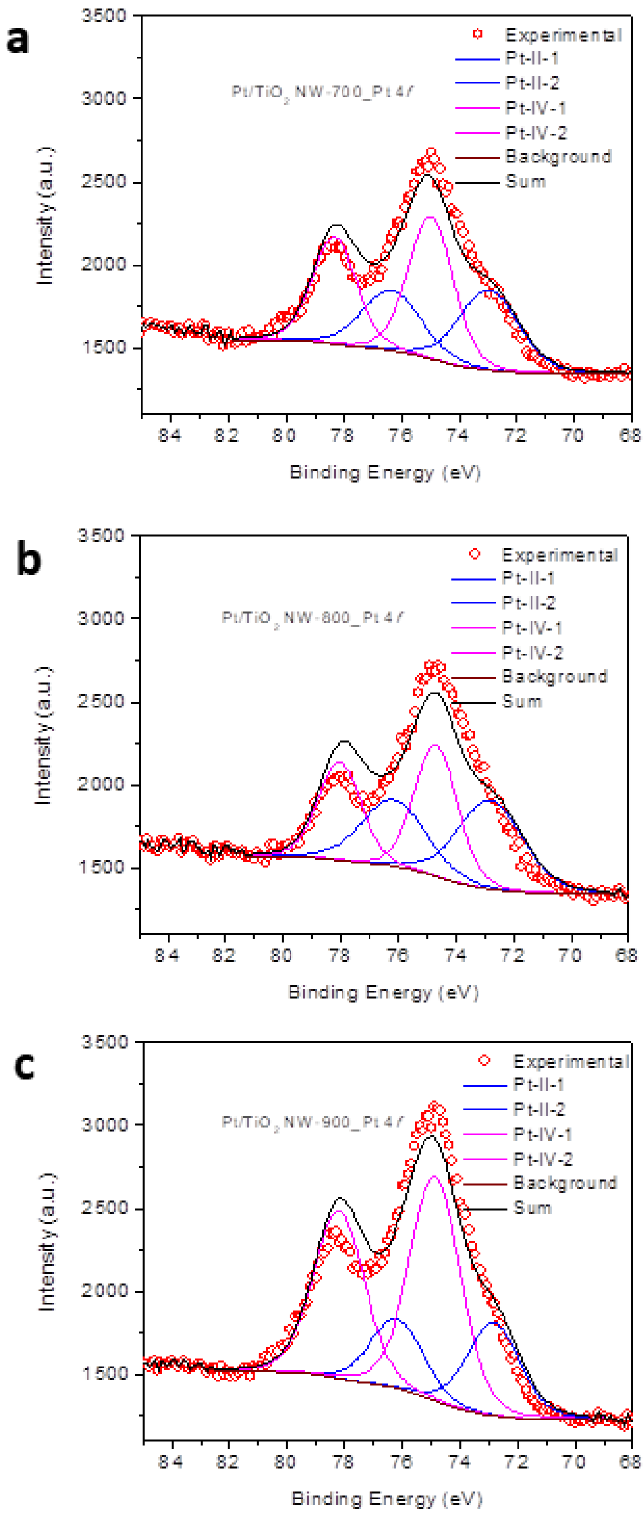

Supplementary Fig. 21 Core Pt 4*f* XPS spectra of Pt_1/TiO_2 NW powder samples with different calcination temperatures (**a**) 700 ^oC; (**b**) 800 ^oC; (**c**) 900 ^oC. Similar to the NW sample calcined at 500 ^oC, Only Pt^{2+} (Pt-II) and Pt^{4+} (Pt-IV) were detected on these samples.

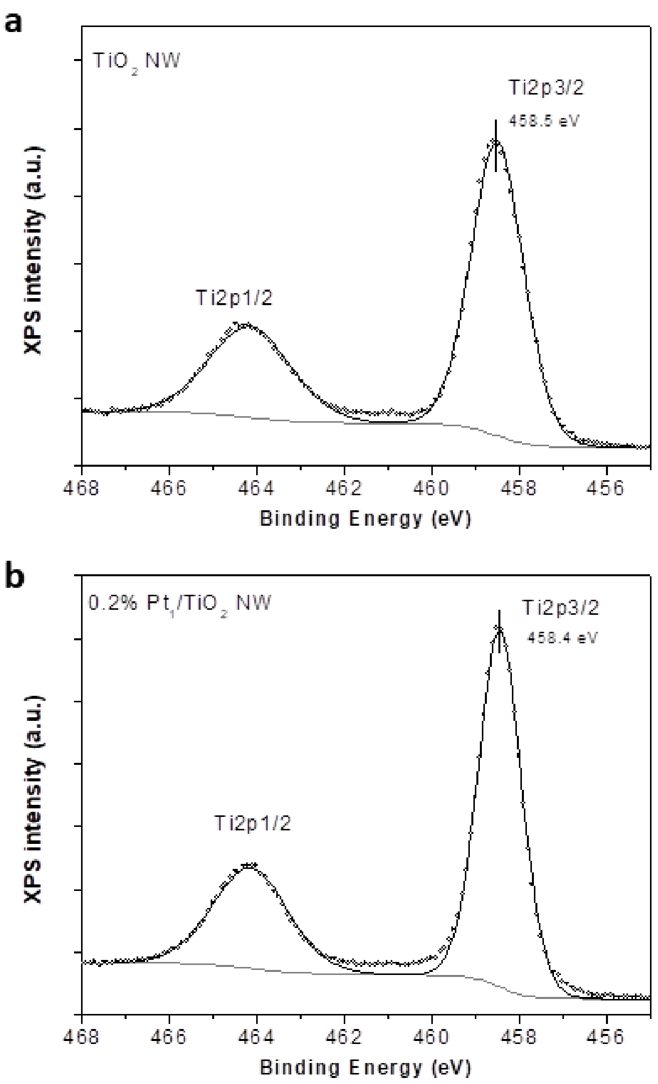

Supplementary Fig. 22 Core Ti 2*p* XPS spectra of (**a**) TiO_2 nanowire (NW) and (**b**) Pt_1/TiO_2 NW powder samples. Only Ti^{4+} was detected on both samples.

Supplementary Table 1 Equivalent Pt loading in Pt/TiO_2 nano-array monolithic samples

Volumetric loading (g L^{-1})	Volumetric loading (g cft^{-1})*	Pt weight loading based on whole core (%)	Pt weight loading based on TiO_2 (%)
0.53	15	0.18	0.77
0.71	20	0.24	1.03
1.73	50	0.58	2.51

*cft: cubic foot (feet)

Supplementary Table 2 Structural parameters of Pt_1/TiO_2 NW and reference samples extracted from the Pt L_3-edge EXAFS fitting. (S_0^2=0.8)[a].

Sample	Atomic scatter	No. of atoms (CN)[b]	Interatomic distance (Å)[c]	Debye-Waller factor (10^{-3}×$Å^2$)[d]	ΔE_0(eV)[e]	*R* factor
Pt-foil	Pt-Pt	12	2.76±0.03	4.4±1.2	6.8	0.003
PtO_2	Pt-O	6	2.01±0.02	3.2±0.9	9.0	0.011
Pt_1/TiO_2 NW	Pt-O	5.5±1.1	1.99±0.02	2.5±0.8	10.3	0.013
Pt NP/TiO_2 NA	Pt-O	0.9±0.2	2.08±0.02	2.1±0.5	17.2	0.006
	Pt-Pt	5.0±1.0	2.73±0.03	7.8±2.9	5.4	

[a] S_0^2 was fixed as 0.8 during EXAFS fitting, based on the known structure of Pt foil.
[b] CN is the coordination number.
[c] Interatomic distance is the bond length between Pt central atoms and surrounding coordination atoms.
[d] Debye-Waller factor is a measure of thermal and static disorder in absorber-scattering distances.
[e]ΔE_0 is the difference between the zero-kinetic energy value of the sample and that of the theoretical model.

Supplementary Table 3 XPS analyses of Pt_1/TiO_2 nanowire powders (NW) after calcination at different temperatures. The XPS data is based on Fig. 2c and supplementary Fig. 21.

NW sample / Pt Oxidation state	Pt_1/TiO_2 NW (500 ^oC)	Pt_1/TiO_2 NW (700 ^oC)	Pt_1/TiO_2 NW (800 ^oC)	Pt_1/TiO_2 NW (900 ^oC)
0 (at. %)	0	0	0	0
II (at. %)	14.84	42.79	50.5	29.99
IV (at. %)	85.15	57.21	49.5	70.01
Pt-II/Pt-IV	0.17	0.75	1.02	0.43
Average Pt-O coordination number *	5.85	5.57	5.50	5.70

*. Single Pt O-coordination number: 6 for Pt-IV, and 5 for Pt-II.

Supplementary Note 1

Cordierite is a magnesium iron aluminium cyclosilicate with a representative chemical formula of $(Mg, Fe)_2Al_3(Si_5AlO_{18})$. Under hydrothermal condition, cordierite substrate can be etched by HCl through the dissolution of Mg, Fe, and Al, leaving behind the mesoporous SiO_2. The cordierite substrate etched in the similarly acidic conditions but without additional titanium precursor displays high surface area, but loses mechanical integrity in the form of the structural collapse due to the excess porosity induced by over-etching. Evidently, the formation of densely packed TiO_2 nano-array layer may have protected the cordierite substrate from aggressive acidic etching during the solvothermal growth.

Supplementary Note 2

The core Pt 4*f* spectra can be deconvoluted to Pt-II and Pt-IV peaks from the XPS data collected from the Pt/TiO_2 NW samples (Fig. 2c and Supplementary Fig. 21). No metallic Pt (Pt-0) peaks were detected in all the samples, as summarized in the Supplementary Table 3. The ratio of Pt-II/Pt-IV increases with increasing calcination temperature from 500 ^{o}C to 800 ^{o}C, indicating the reduced oxygen ion coverage on top of the single Pt atoms on the TiO_2 nanowire surfaces, with the average Pt O-coordination number decreasing from 5.85, 5.57, to 5.50. No further increase of the ratio of Pt-II/Pt-IV was observed when the temperature increases to 900 ^{o}C, with an average Pt O-coordination number of 5.70 (Supplementary Table 3). This result is consistent with the Pt L_3-edge EXAFS fitting result shown in the Supplementary Table 2.

Oxygen vacancies in TiO_2 can be monitored through monitoring Ti^{3+} since the electrons left in the vacancy are transferred to the neighboring Ti atoms, forming two Ti^{3+} cations per one oxygen vacancy. As shown in Supplementary Fig. 22, the core Ti2*p* XPS spectra of both TiO_2 NW powder and Pt_1/TiO_2 NW powder show only peaks at 464.3 eV and 458.4 - 458.5 eV, corresponding to the Ti2*p*1/2 and Ti2*p*3/2 of Ti^{4+}. Since XPS is a surface sensitive technique with a penetration depth of ~2-10 nm, this means that there is no surface Ti^{3+} and oxygen vacancies. This result is expected since the synthesis process of Pt_1/TiO_2 NA and NW involves a calcination process at high temperature, typically at 500 ^{o}C, in which surface Ti^{3+}, if existed, can be easily oxidized to Ti^{4+}. This observation is consistent with the results obtained in the NA and NW samples calcined at the different temperatures (700 ^{o}C, 800 ^{o}C, and 900 ^{o}C, respectively).

SUPPLEMENTARY REFERENCES

1. Hoang, S., Guo, S., Hahn, N.T., Bard, A.J., & Mullins, C.B., Visible Light Driven Photoelectrochemical Water Oxidation on Nitrogen-Modified TiO_2 Nanowires. *Nano Lett.* 12 (1), 26-32 (2012).
2. Feng, X. *et al.*, Vertically Aligned Single Crystal TiO_2 Nanowire Arrays Grown Directly on Transparent Conducting Oxide Coated Glass: Synthesis Details and Applications. *Nano Lett.* 8 (11), 3781-3786 (2008).
3. Avila, M.S. *et al.*, Effect of V_2O_5 Loading on Propane Combustion over $Pt/V_2O_5–Al_2O_3$ Catalysts. *Catal. Lett.* 134 (1), 118-123 (2010).
4. Kresse, G., Furthmüller, J., Efficiency of ab-initio Total Energy Calculations for Metals and Semiconductors using A Plane-wave Basis Set, *Comput. Mater. Sci.*, 6, 15-50 (1996).
5. Kresse, G., Furthmüller, J., Efficient Iterative Schemes for ab initio Total-energy Calculations using A Plane-wave Basis Set, *Phys. Rev. B*, 54, 11169 (1996).
6. Perdew, J.P., Burke, K., Ernzerhof, M., Generalized Gradient Approximation Made Simple, *Phys. Rev. Lett.*, **77**, 3865 (1996).
7. Grimme, S., Semiempirical GGA-type Density Functional Constructed with A Long-range Dispersion Correction, *J. Comp. Chem.*, 27, 1787-1799 (2006).
8. Henkelman, G., Uberuaga, B.P., Jónsson, H., A Climbing Image Nudged Elastic Band Method for Finding Saddle Points and Minimum Energy Paths, *J. Chem. Phys.*, 113, 9901-9904 (2000).
